# Immunomodulatory effects of Yang He decoction on cyclophosphamide-induced immunosuppression in mice: restoration of immune organ integrity and cytokine balance

**DOI:** 10.3389/fphar.2026.1805534

**Published:** 2026-06-16

**Authors:** Yubo Wang, Qianqian Xu, Wenliang Xu, Yanping Wang, Yu’na Wang, Mimi Qin, Juanjuan Dai, Songlin Zhang

**Affiliations:** 1 Department of Thoracic Surgery, Binzhou Medical University Hospital, Binzhou, China; 2 Shandong Binzhou Animal Science and Veterinary Medicine Academy, Binzhou, China; 3 School of Breeding and Multiplication, Hainan University, Sanya, China; 4 College of Biological and Environmental Engineering, Shandong University of Aeronautics, Binzhou, China; 5 Department of Pathology, Binzhou Medical University Hospital, Binzhou, China; 6 Clinical Laboratory Center, Binzhou Medical University Hospital, Binzhou, China

**Keywords:** cyclophosphamide, cytokines, immunomodulation, immunosuppression, splenic lymphocyte, Yang He decoction

## Abstract

**Objective:**

To evaluate the protective effects and potential mechanisms of Yang He decoction (YHD), a classic traditional Chinese medicine formula, against cyclophosphamide (CTX)-induced immunosuppression in mice.

**Methods:**

An immunosuppression model was established in Kunming mice *via* intraperitoneal injection of CTX (80 mg/kg). The mice were then treated with low, medium, or high doses of YHD (50, 100, or 200 mg/kg) or Astragalus polysaccharide (APS, 24 mg/kg) as a positive control for 15 consecutive day. Body weight changes, immune organ (spleen and thymus) index and histopathology, splenic lymphocyte proliferation and cell cycle distribution, and serum levels of cytokines (IL-1β, IFN-γ, TNF-α, IL-6, and IL-10) were systematically assessed.

**Results:**

YHD treatment significantly ameliorated CTX-induced immunosuppression. The medium dose (100 mg/kg) increased body weight by 58.5% (vs. 17.3% in the APS group), restored the spleen index to 9.41 ± 0.94 (0.52-fold vs. Model group 1.8 ± 0.3), and enhanced ConA-stimulated lymphocyte proliferation (27.03% ± 0.92% vs. Model group 10.60% ± 4.78%, *P* < 0.01). Cell cycle analysis revealed that the percentage of S-phase cells increased from 4.13% to 13.47% in the YHDH group. YHD also rebalanced cytokines: the concentration of IL-1β increased from 25.73 to 77.10 pg/mL, while IL-10 decreased from 222.83 to 20.77 pg/mL. Histopathology confirmed restoration of organ integrity without necrosis.

**Conclusion:**

The multiherbal formula YHD confers substantial protection against CTX-induced immunosuppression. Its mechanisms are associated with the restoration of immune organ integrity, promotion of lymphocyte activity, and rebalancing of the cytokine profile. These findings provide a pharmacological basis for the traditional use of YHD and support its potential as an adjuvant therapy for managing chemotherapy-related immunosuppression.

## 1 Introduction

Yang He decoction (YHD) is a classical multiherbal formula in traditional Chinese medicine (TCM) that was first documented in the Qing dynasty Life-saving Handbook for Diagnosing and Treating External Ailments (Waike Zhengzhi Quansheng Ji). Unlike seven medicinal plants, YHD is traditionally prescribed to “dispel cold and promote circulation” and “improve yang to replenish blood” for conditions such as Yinju (akin to pyogenic osteomyelitis). From a modern biomedical perspective, its therapeutic principle of “strengthening the body’s resistance to eliminate pathogenic factors” aligns closely with the concept of immunomodulation (Zheng et al., 2017).

In contemporary research, YHD has shown promise as an adjuvant therapy with immunomodulatory effects in various conditions, including breast cancer (Zhang et al., 2022; Mao et al., 2018), ankylosing spondylitis (Xu et al., 2020), and lung cancer (Wu L et al., 2021). Mechanistic studies have indicated that it can regulate cytokine levels (Zeng and Yang, 2017), mitigate immune disorders in asthmatic mice (Li et al., 2022), and ameliorate autoimmune thyroiditis in rats by modulating the NLRP3 inflammasome (Ma et al., 2021). These findings underscore the critical role of immunomodulation in the clinical efficacy of YHD, particularly in immunocompromised settings.

However, despite its traditional use and emerging applications, a significant gap remains in the systematic evaluation of the immunoenhancing potential of YHD in a state of generalized immunosuppression. CTX, one of the most successful and widely used antineoplastic drugs (Emadi et al., 2009), is also associated with severe immunosuppression, exerts its immunosuppressive effects by depleting lymphoid progenitor cells; damaging DNA structure; disrupting the proliferation and differentiation of macrophages, T cells, and B cells; and impairing humoral and cellular immune responses (Cruz-Chamorro et al., 2007). To assess the immunoenhancing potential of YHD in a clinically relevant setting, the CTX-induced immunosuppressed mouse model has become a standard platform in TCM immunopharmacology research, as it reliably replicates the hallmarks of chemotherapy-induced immune impairment, including reduced immune organ index, lymphocytopenia, and dysregulated cytokine production. Importantly, a validated intermittent dosing regimen—80 mg/kg CTX administered intraperitoneally on days 1, 2, 3, 7, and 11—has been shown to induce a stable and reproducible subacute immunosuppressive state, effectively mimicking the cumulative immunosuppressive effects observed in clinical multidrug chemotherapy cycles while allowing sufficient time for subsequent treatment observation (Yang et al., 2023; Liu et al., 2015). This model has been widely adopted in similar pharmacological studies of natural immunomodulators (Mao et al., 2018), thus serving as a relevant and robust platform for assessing the immune-restorative properties of YHD.

Therefore, this study aims to provide an ethnobotanical pharmacological validation of YHD. Using a CTX-induced immunosuppressed mouse model, we investigated the effects of YHD on key immune parameters, such as body weight dynamics, immune organ (spleen and thymus) histopathology, splenic lymphocyte proliferation, the cell cycle, and serum cytokine profiles. Our findings elucidate the immunomodulatory mechanisms of YHD and support its potential development as a complementary therapy for managing chemotherapy-induced immunosuppression.

## 2 Materials and methods

### 2.1 Ethics statement

The animal study protocol was reviewed and approved by the Experimental Animal Ethics Committee of Binzhou Medical University Hospital (Approval Number: 20220128-66). All procedures were conducted in strict accordance with the institutional guidelines and the ARRIVE guidelines 2.0 (Percie du Sert et al., 2020).

### 2.2 Plant materials and preparation of YHD

The seven herbal components of YHD were authenticated according to the Pharmacopeia of the People’s Republic of China (2020 edition) (Commission, 2020). Voucher specimens (voucher nos. 2112YHD01 to 2112YHD07) have been deposited at our institute for future reference. The formulation ratios are detailed in Supplementary Table S1.

To prepare the extract, a total of 165 g of crude herb (at the specified ratio) was subjected to reflux extraction twice with 1,650 mL of distilled water (100 °C, 1 h per extraction). The combined supernatants were centrifuged (1,500 × g), concentrated under reduced pressure at 60 °C to a density of 1.5 g/mL, and finally lyophilized. The lyophilized powder was obtained with a yield of 50.76% ± 0.56% and stored at −20 °C until use.

### 2.3 HPLC analysis of YHD chemical markers

The chemical profile of YHD was characterized *via* an Agilent 1200 series HPLC system equipped with a G1260 VWD detector and an InfinityLab Poroshell 120 EC-C18 column (2.1 × 100 mm, 2.7 μm). The column temperature was maintained at 40 °C. The mobile phase consisted of (A) acetonitrile and (B) 0.1% phosphoric acid with the following gradient: 5%–20% A (0–50 min), 20%–80% A (50–60 min), 80%–5% A (60–65 min), and 5% A (65–75 min). The flow rate was 0.35 mL/min, the injection volume was 3 μL, and detection was performed at 210 nm.

**FIGURE 1:**
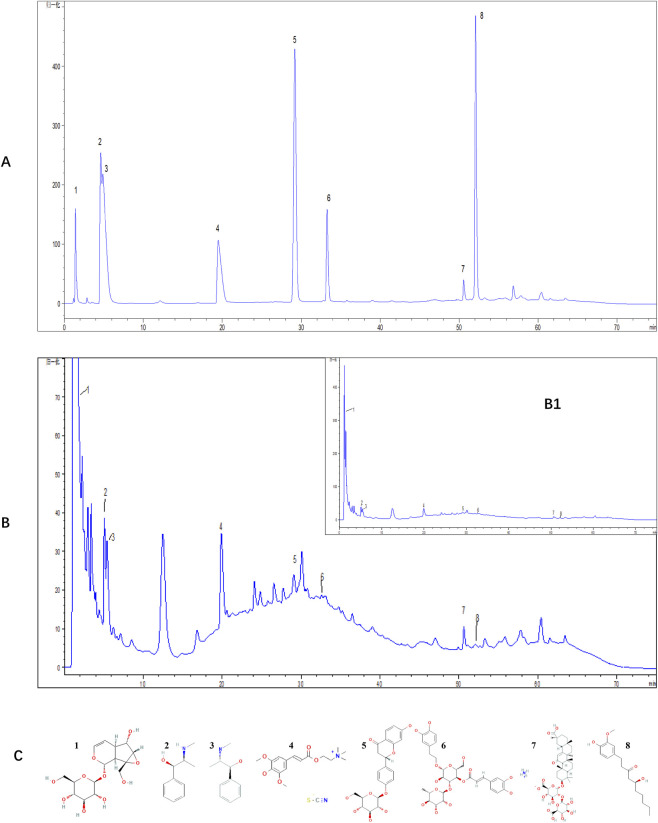
HPLC chromatograms of representative active components in YHD. **(A)** Chromatograms of eight reference standards; **(B)** main chromatogram of YHD; (B1) whole chromatogram of YHD; **(C)** chemical structures of the eight standards. 1, catalpol; 2, ephedrine; 3, pseudoephedrine; 4, sinapine thiocyanate; 5, liquiritin; 6, acteoside; 7, glycyrrhizic acid ammonium salt; 8, 6-gingerol.

Reference standards for major constituents from the component herbs, including catalpol and acteoside *(Rehmannia glutinosa*), ephedrine and pseudoephedrine (*Ephedra sinica*), sinapine thiocyanate (*Sinapis alba*), liquiritin and glycyrrhizic acid ammonium salt (*Glycyrrhiza uralensis*), and 6-gingerol (*Zingiber officinale*), were purchased with purities >98%. Sample and standard solutions were prepared in 0.1% phosphoric acid and filtered (0.22 μm) prior to injection. A representative HPLC chromatogram is presented in Figure 1.

### 2.4 Animals and experimental design

One hundred twenty specific pathogen-free (SPF) Kunming mice (20 ± 2 g, half male and half female) were purchased from Ji’nan Peng Yue Laboratory Animal Breeding Co., Ltd. (SCXK (Lu) 2019-0003). The mice were housed under standard conditions (25 °C, 12/12 h light/dark cycle) with free access to food and water and acclimatized for 1 week prior to experimentation.

After acclimatization, the mice were randomly divided into two cohorts: a normal control group (Control, n = 20) and a cohort for immunosuppression induction (n = 100). Immunosuppression was induced by intraperitoneal (i.p.) injection of CTX (80 mg/kg) on days 1, 2, 3, 7, and 11, a subacute regimen designed to mimic the cumulative immunosuppression observed in clinical multicycle chemotherapy (Cui et al., 2023). The control group received injections of equal volumes of normal saline.

On day 12, the CTX-treated mice were further randomized into five groups (n = 20 per group): the model group (Model), the positive control group (APS, 24 mg/kg), and three YHD treatment groups, which received low (YHDL, 50 mg/kg), medium (YHDM, 100 mg/kg), and high (YHDH, 200 mg/kg) doses. Within each group, the 20 mice were further subdivided: 10 mice were randomly allocated for the peritoneal macrophage phagocytosis assay, and the remaining 10 mice were used for histopathological examination, splenic lymphocyte proliferation assays, cell cycle analysis, and serum cytokine measurement. Treatments were administered daily by oral gavage for 15 consecutive days. The control and model groups received an equal volume of distilled water. The treatment schedule is summarized in Table 1.

**TABLE 1:** Treatments in each group.

Groups	Intraperitoneal injection (on d 1, 2, 3, 7, and 11)	Oral administration (from d 12 to d 26, daily)
Control	Normal saline (NS)	NS
Model	CTX, 80 mg/kg	NS
APS	CTX, 80 mg/kg	APS, 24 mg/kg
YHDL	CTX, 80 mg/kg	YHD, 50 mg/kg
YHDM	CTX, 80 mg/kg	YHD, 100 mg/kg
YHDH	CTX, 80 mg/kg	YHD, 200 mg/kg

APS (24 mg/kg) was selected on the basis of previous studies demonstrating immunomodulatory effects at 20–40 mg/kg in mice (Cui et al., 2023). The YHD dose was determined by converting the clinical daily dose for a 60 kg adult (approximately 54 g crude herb, 0.9 g/kg/day) (Fei, 2011; Wang et al., 2016) to a mouse equivalent dose using a body surface area conversion factor of 9.01 (Zhi et al., 2025), yielding a medium dose of 100 mg/kg extract. Low and high doses were set at 0.5× and 2× the medium dose, respectively, to explore the dose‒response relationship (Chen et al., 2011). This dose range is consistent with that used in previous *in vivo* studies by YHD (He et al., 2023).

### 2.5 Evaluation of general health and immune organ index

The mice were weighed daily to determine the rate of body weight change, and their general physiology was concurrently observed for a period of 26 days, following formula 1:

$$\text{Change rate of body weight}=\frac{W_{x}-W_{1}}{W_{1}}\times 100\%$$ (1)

W_x_: body weight on the experimental day; W_1_: body weight on the first day

On d 27, all the mice were euthanized. The spleen and thymus were excised, cleared of connective tissue, and weighed. The organ index was calculated according to formula 2. Peyer’s patches in the small intestine were counted under a dissecting microscope.

$$Index\;\left(\text{mg}/g\right)=\frac{\text{Weight}\;\text{of}\;\text{thymus}\;\text{or}\;\text{spleen}}{\text{Body}\;\text{weight}}$$ (2)

### 2.6 Phagocytic function of peritoneal macrophages

After a 12-h fast following the last administration, the mice were injected i.p. with 1 mL of 10% chicken red blood cells (CRBCs). Thirty minutes later, the peritoneal exudate was collected by lavage with 2 mL of normal saline. Smears were prepared, fixed, and stained with Giemsa solution. The phagocytic rate and phagocytic index were determined *via* light microscopy as Formulas 3, 4:

$$Phagocytic\text{ rate}=\frac{\text{Macrophages}\;\text{that}\;\text{phagocytized}\;\text{chicken}\;\text{red}\;\text{cells}}{\text{Total}\;\text{macrophages}}\times 100\%$$ (3)

$$Phagocytic\text{ index}=\frac{\text{Chicken}\;\text{red}\;\text{cells}\;\text{phagocyted}\;\text{by}\;\text{macrophages}}{\text{Total}\;\text{macrophages}}$$ (4)

### 2.7 Histopathological examination of the spleen and thymus

Spleen and thymus tissues were fixed in 10% neutral buffered formalin, embedded in paraffin, sectioned (4 μm thickness), and stained with hematoxylin and eosin (H&E). Histopathological changes were observed and imaged under a light microscope (Jayaraman and Variyar, 2021; Wang et al., 2020).

### 2.8 Splenic lymphocyte proliferation assay

A single-cell suspension of splenocytes was prepared aseptically, and erythrocytes were lysed in ACK buffer. The cells were resuspended in RPMI-1640 medium supplemented with 10% FBS and antibiotics at a density of 5 × 10^6^ cells/mL, seeded into 96-well plates (100 μL/well) and stimulated with concanavalin A (ConA, 5 μg/mL) or lipopolysaccharide (LPS, 10 μg/mL) for 72 h at 37 °C in 5% CO_2_. Cell proliferation was assessed *via* the WST-8 assay, with the absorbance measured at 450 nm (Wang et al., 2016). The proliferation index was calculated as Formula 5:

$$Cell\text{ viability }\left(\%\right)=\frac{{\text{OD}}_{1}-{\text{OD}}_{0}}{{\text{OD}}_{0}}\times 100\%$$ (5)

OD_1_: Absorbance value under stimulation, OD_0_: Absorbance value without stimulation

Cell proliferation was measured *via* the WST-8 assay as a surrogate for viable cell number. To provide more direct evidence of cell division, cell cycle analysis was performed by flow cytometry as described in Section 2.9.

### 2.9 Cell cycle analysis of splenic lymphocytes

Splenic lymphocytes were fixed in ice-cold 70% ethanol overnight at 4 °C and washed with PBS and stained with a propidium iodide solution containing RNase (KeyGEN BioTECH, China) for 45 min in the dark. Cell cycle distribution was analyzed using a flow cytometer (BD Biosciences, USA), and the proliferation index (PI) (Jayaraman and Variyar, 2021) was calculated acccording to formula 6. A hierarchical gating strategy was applied: cells were first gated by side scatter area (SSC-A) vs. forward scatter area (FSC-A) to exclude debris and select intact cells (P1); doublets were then excluded by FSC-A vs. FSC-H gating (P2); finally, cell cycle phases (G_0_/G_1_, S, and G_2_/M) were determined by analyzing PI fluorescence area (PI-A) histograms of the single-cell population using automated curve fitting.

$$PI=\frac{Cell\;\text{number}\;\text{of}\;S\;\text{stage}+\text{Cell}\;\text{number}\;\text{of}\;\left(G_{2}+M\right)\;\text{stage}}{\text{Total}\;\text{cells}}\times 100$$ (6)

### 2.10 Measurement of serum cytokine levels

Blood samples were collected from the orbital plexus, and the serum was separated by centrifugation (1,000 × g, 20 min). The concentrations of IL-1β, IL-6, IL-10, TNF-α, and IFN-γ were quantified *via* specific commercial ELISA kits (ABclonal, China) according to the manufacturer’s instructions. The absorbance was read at 450 nm, and the cytokine concentrations were interpolated from the respective standard curves.

### 2.11 Statistical analysis

All the data are expressed as the means ± standard deviations (SDs). Statistical analyses were performed using SPSS software (version 20.0). The normality of the data distribution was first assessed using the Shapiro‒Wilk test. For normally distributed data, comparisons among multiple groups were conducted using one-way analysis of variance (ANOVA). When the ANOVA results indicated statistically significant differences, Tukey’s honestly significant difference (HSD) *post hoc* test was applied for pairwise comparisons between groups, which were inherently corrected for multiple comparisons. A value of *P* < 0.05 indicated statistical significance. All the statistical tests and significance indicators are specified in the respective figure and table legends.

## 3 Results

### 3.1 Effects of YHD on the general physiological state

Mice that received CTX injection presented additional symptoms, including hypotrichosis and lackluster fur, hypothermia upon touch, reduced activity, and decreased food intake, confirming the experimental model (Li et al., 2021). In contrast, the mice in the control group displayed normal behavior. Compared with those in the model group, the conditions of the mice in the APS and YHD groups improved, with only slight hypotrichosis, normal body temperature, and regular food consumption.

**FIGURE 2:**
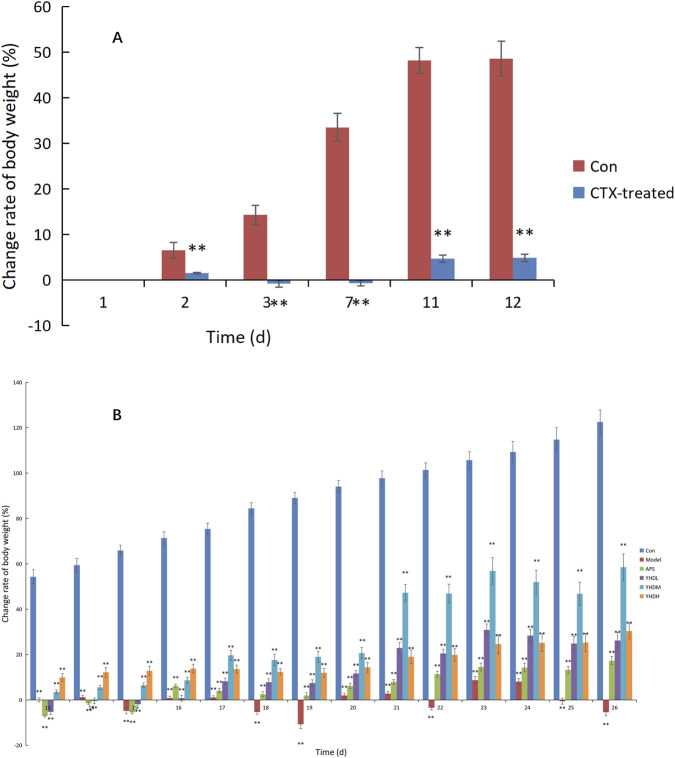
Effects of YHD and APS on body weight in CTX-treated mice. CTX was administered intraperitoneally on days 1, 2, 3, 7, and 11. YHD or APS was administered orally from day 12 to day 26. **(A)** Body weight growth rates from day 1 to day 12 in the control group and the CTX-treated cohort. **(B)** Body weight growth rates from day 13 to day 26 in mice receiving APS, YHD or vehicle. The data are presented as mean ± SD (n = 20 per group). Con: control group; Model, CTX-treated mice given vehicle; APS: CTX-treated mice given 24 mg/kg APS; YHDH, YHDM, YHDL, CTX-treated mice given 200, 100 and 50 mg/kg YHD, respectively. ^*^ *P <* 0.05, ^**^ *P <* 0.01 vs. control group.

The body weight growth rate of mice from d 1 to d 12 in both the control group and the group treated with CTX is shown in Figure 2A, demonstrating the impact of CTX on body weight. The results indicate that the mice in the control group experienced continuous growth, whereas those in the CTX group exhibited minimal growth, highlighting a significant difference between the two groups. The body weight growth rate of the mice from d 13 to d 26 following TCM treatment is shown in Figure 2B. Specifically, during the initial phase of TCM treatment after model induction (from d 13 to d 18), there was a slight increase in the growth rate; however, during the later period of TCM treatment following model establishment (from d 19 to d 26), there was a notable increase in the growth rate, particularly among the mice in the YHDM group, whose growth rate was significantly greater than that in the other groups. Notably, the growth rate of the mice in the APS group consistently lagged behind that of the YHD groups throughout the entire experimental period. On d 13, the weight increase rate of the APS group was −7.1%, whereas the weight increase rates were −5.3%, 3.5%, and 9.9% in the low, middle, and high YHD groups, respectively. On d 19, the APS group had a growth rate of 2.0%, whereas the low, middle, and high YHD groups had rates of 7.5%, 19.0% and 12.0%, respectively. By d 26, the APS group had a growth rate of 17.3%, whereas the low, middle, and high YHD groups had rates of 26.2%, 58.5%, and 30.4%, respectively. The actual body weight measurements for all the mice at each time point are provided in Supplementary Table S2.

### 3.2 The impact of YHD on the index of the spleen, thymus and Peyer’s patch number

**FIGURE 3:**
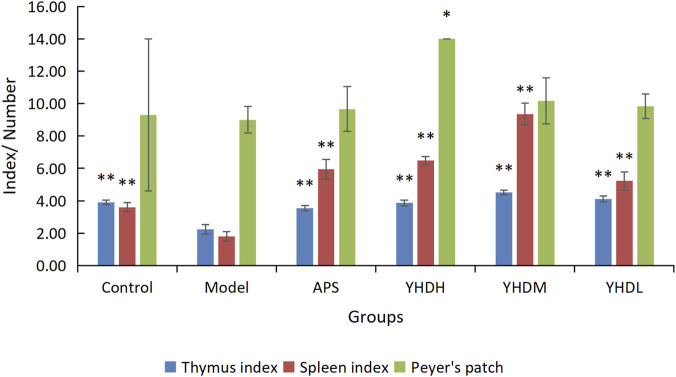
Effects of YHD on spleen and thymus index, and Peyer’s patch number in CTX-treated mice. CTX treatment significantly reduced the spleen and thymus index and Peyer’s patch number compared with the control group. YHD treatment, especially the medium dose (YHDM, 100 mg/kg), markedly increased these parameters. The YHDM group had the highest spleen index (9.41 ± 0.94) and Peyer’s patch count (14.0 ± 0.0). Data are presented as mean ± SD (n = 20 per group). ^*^ *P <* 0.05, ^**^ *P <* 0.01 vs. model group.

Compared with the model groups (1.8 ± 0.3), the other groups presented a higher spleen index (*P* < 0.05 or *P* < 0.01) (Figure 3). The spleen index of the APS, YHDL, YHDM, and YHDH groups were 3.3, 3.6, 5.2, and 2.9 times greater than those of the model group, respectively. The YHDM group had the highest spleen index (9.41 ± 0.94), with some individual variation, reflecting biological variability in the response to treatment during recovery from CTX-induced immunosuppression. Thymus index significantly improved in the APS, YHDL, YHDM, and YHDH groups and were 1.6, 1.86, 2.0, and 1.77 times greater than those in the model group, respectively. In the model group (9.0 ± 2.1), the Peyer’s patch number was slightly lower than that in the control group (9.3 ± 4.7), significantly lower than that in the YHDH group (14.0 ± 0.0) (*P* < 0.05), and slightly lower than that in the other groups, which is consistent with earlier reports (Zhou et al., 2001). The relatively large standard deviation in the control group (9.3 ± 4.7) reflects normal biological variation in the number of Peyer’s patches among individual mice, as these lymphoid aggregates are distributed discontinuously along the small intestine, and their count can vary naturally between animals (Zhou et al., 2001). Obviously, CTX can decrease the number of Peyer’s patches, thereby impairing intestinal immunity. However, YHD has the potential to mitigate this detrimental effect and ameliorate it. YHD may enhance immune function impairment by mitigating the reduction in size and volume of the spleen and thymus and improving the number of Peyer’s patches.

### 3.3 Effects of YHD on the pathology of the spleen and thymus in mice with CTX-induced immunosuppression

**FIGURE 4:**
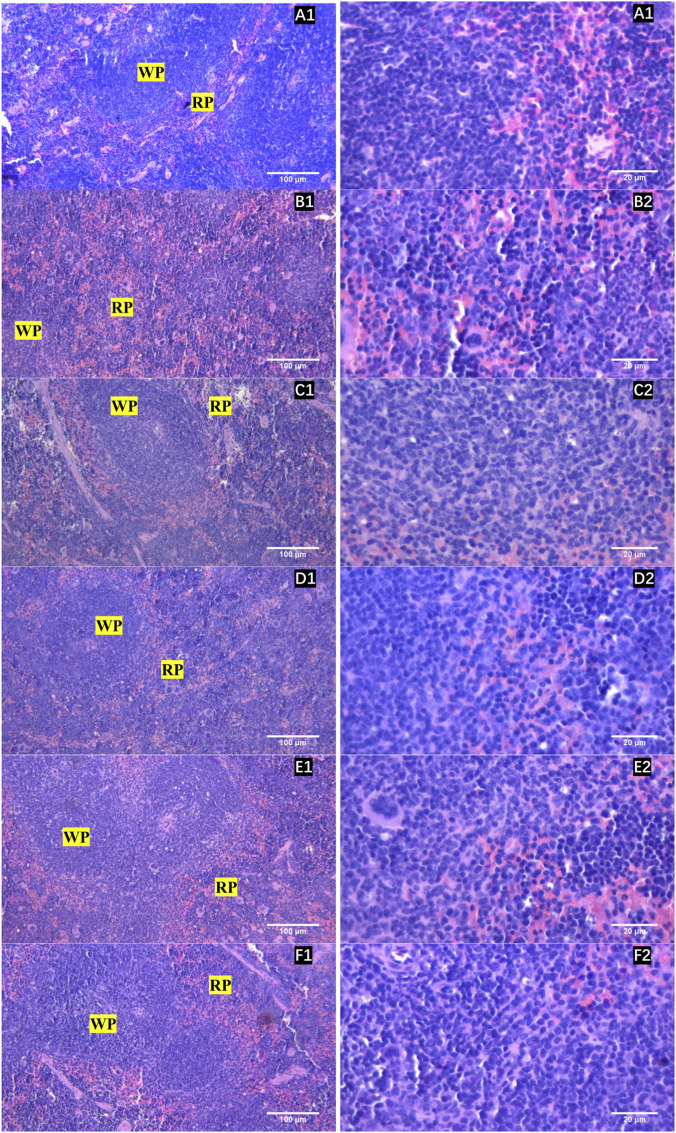
Histopathology of the spleen (hematoxylin and eosin staining). The control group **(A)** shows well-preserved splenic architecture with clear boundaries between red pulp (RP) and white pulp (WP), and densely arranged lymphocytes. The model group **(B)** exhibits disorganized structure, loss of clear WP/RP demarcation, and reduced lymphocytes with disordered arrangement. YHD-treated groups **(D–F)** and APS group **(C)** show increased lymphocyte proliferation and improved WP/RP boundary Arabic numbers “1” and “2” indicate ×100 and ×400 magnification, respectively.

Histological analysis *via* HE staining revealed that the spleen in the control group presented a well-preserved structure, with distinct boundaries between the red pulp (RP) and white pulp (WP) and that the lymphocytes were closely arranged. On the other hand, the splenic corpuscles in the model group exhibited disorganized organization, a lack of clear demarcation between RP and WP, and a reduced number of lymphocytes with a disordered arrangement. Compared with the model group, the YHD and APS groups presented increased lymphocyte proliferation and an improved boundary between WP and RP (Figure 4).

**FIGURE 5:**
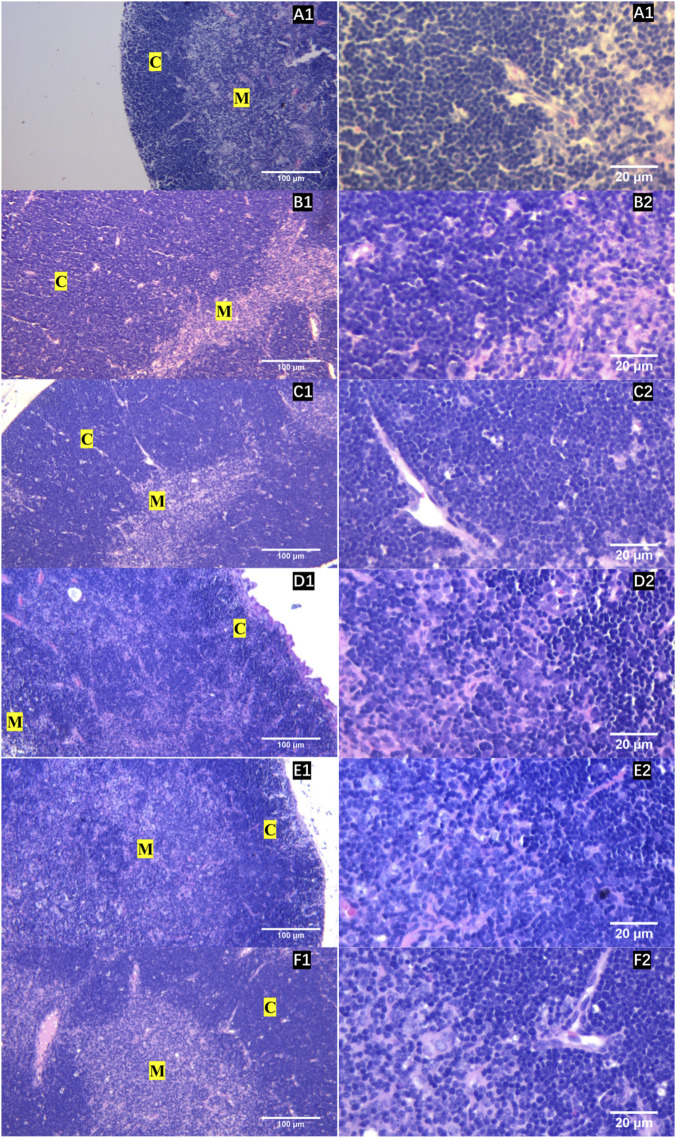
Histopathology of the thymus (hematoxylin and eosin staining). The control group **(A)** shows a normal thymic cortex (CO) densely packed with proliferating lymphocytes. The model group **(B)** displays fewer cortical lymphocytes, an increased number of reticular cells, cortical malformation, shrunken nuclei, and cracked thymocytes detached from adjacent tissue. These pathological changes are attenuated in YHD-treated groups **(D–F)** and the APS group **(C)**. ME: medulla. Arabic numbers “1” and “2” indicate ×100 and ×400 magnification, respectively.

In the control group, the outer cortex of the thymus was filled with proliferating lymphocytes. Conversely, the model group displayed fewer lymphocytes in the cortex but instead presented a large quantity of reticular cells. Additionally, the cortex exhibited malformation, shrunken nuclei, and cracked thymus cells, which were disconnected from adjacent tissues in multiple zones. These phenomena decreased in the group treated with YHD (Figure 5).

YHD promotes the restoration of both innate and adaptive immunity by alleviating the pathological changes in the structures of the spleen and thymus induced by CTX, as further supported by functional assays demonstrating increased lymphocyte proliferation, macrophage phagocytosis, and cytokine modulation.

### 3.4 Effects of YHD on ConA- and LPS-induced splenic lymphocyte proliferation

**FIGURE 6:**
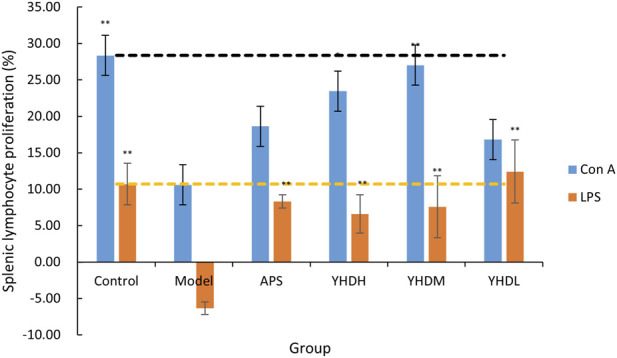
Effects of YHD on ConA- or LPS-induced splenic lymphocyte proliferation in CTX-treated mice. Splenic lymphocytes were stimulated with ConA (5 μg/mL) or LPS (10 μg/mL) for 72 h, and proliferation was measured by WST-8 assay. Data are presented as mean ± SD (n = 10 per group). ^*^ *P <* 0.05 and ^**^ *P <* 0.01 vs*.* model group. The dashed horizontal line indicates the mean value of the control group. Control, normal saline-treated mice; Model, CTX-treated mice given vehicle; APS, CTX-treated mice given 24 mg/kg APS; YHDL, YHDM, YHDH: CTX-treated mice given 50, 100, or 200 mg/kg YHD, respectively.

In this study, lymphocyte proliferation was significantly greater in the control group than in the model group (Figure 6, *P* < 0.01), which aligns with the findings reported by Bear (Bear, 1986). The splenic lymphocytes in the model group (−6.33% ± 0.85%) exhibited a lack of proliferation following LPS stimulation, indicating their weakened state after CTX treatment. Compared with those in the model group, the proliferation of splenic lymphocytes in the APS (8.31% ± 0.91%), YHDH (6.60% ± 2.61%), and YHDM (7.58% ± 4.27%) groups increased following LPS stimulation, with the YHDL group (12.42% ± 4.34%) exhibiting the strongest effect. In response to ConA stimulation, compared with the model group (10.60% ± 4.78%), all the treatment groups exhibited increased lymphocyte proliferation. The YHDM group (27.03% ± 0.92%) showed the greatest increase, which was comparable to that of the control group (28.35% ± 3.90%) and significantly greater than that of the model group (*P* < 0.01). Compared with the model group, the APS group (18.63% ± 3.36%) also showed significant improvement (*P* < 0.01). These results demonstrate that YHD treatment, particularly at a medium dose (100 mg/kg), can effectively restore splenic lymphocyte proliferation in CTX-immunosuppressed mice.

### 3.5 Analysis of the cell cycle

**FIGURE 7:**
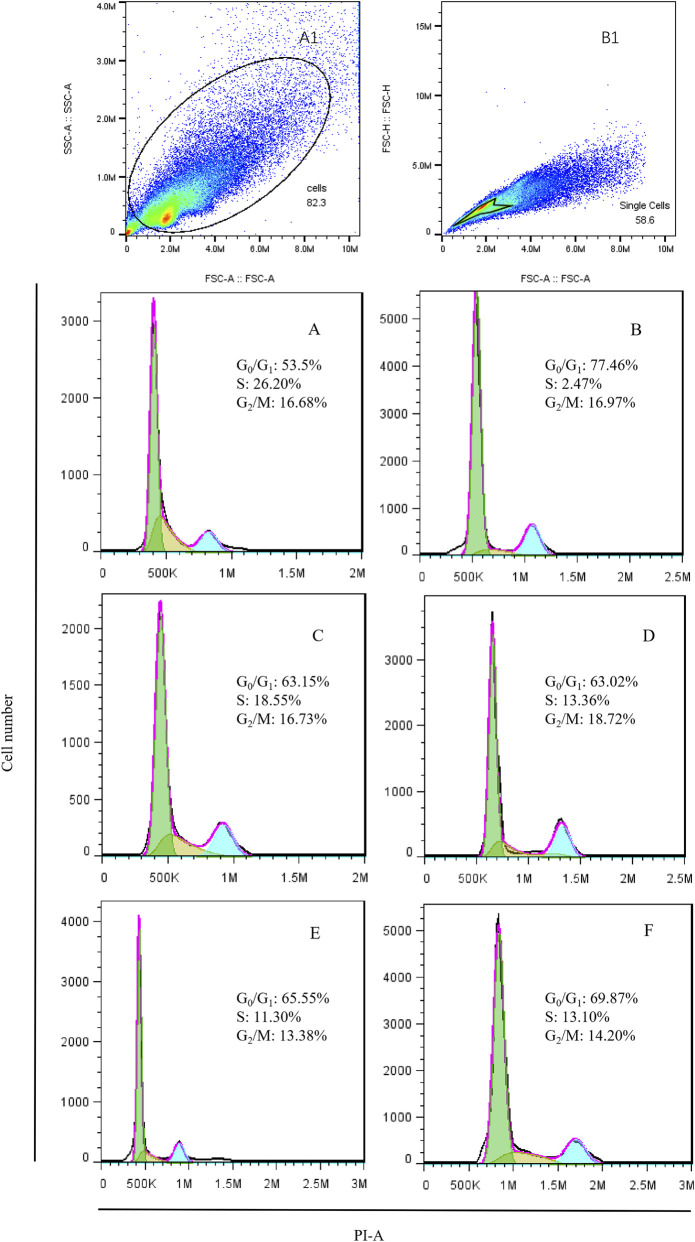
Flow cytometry gating strategy and cell cycle analysis of mouse splenic lymphocytes. **(A**1**)** FSC-A vs. SSC-A dot plot showing selection of intact cells (P1) after debri exclusion. **(B**1**)** FSC-A vs. FSC-H dot plot showing single-cell gating (P2) after doublet exclusion. **(A–F)** Representative PI-A histograms of the single-cell population (P2) from each experimental group, with the percentages of cells in G_0_/G_1_, S, and G_2_/M phases indicated. Data were analyzed using automated curve fitting. Control, normal saline-treated mice; Model, CTX-treated mice given vehicle; APS, CTX-treated mice given 24 mg/kg APS; YHDL, YHDM, YHDH, CTX-treated mice given 50, 100, or 200 mg/kg YHD, respectively.

Analysis of the cell cycle confirmed the stimulating effect of YHD on DNA synthesis in immunosuppressed mice. Compared with those in the control group, the percentages of cells in the S phase and the PI in the model group were notably lower (*P* < 0.01 and *P* < 0.05), especially the percentages of cells in the S phase, which were significantly lower than those in the other groups, which aligns with the findings of a previous study (Wang and Wang, 1994). Compared with the control treatment, the administration of APS resulted in a modest decrease in the number of cells in S phase but a significantly greater increase in the number of cells in S phase. Compared with the model group, the YHD group presented a greater number of S-stage cells and a greater PI (Figure 7; Table 2). These findings suggest that YHD treatment can moderate the cell cycle of splenic lymphocytes in mice with CTX-induced immunosuppression, although its effect is slightly inferior to that of APS.

**TABLE 2:** Effects of YHD administration for 15 days on the splenic lymphocyte cell cycle in CTX-induced immunosuppressed mice (n = 10).

Groups	G_0_/G_1_ stage (%)	S stage (%)	G_2_/M stage (%)	PI
Control	57.90 ± 1.52	24.43 ± 1.84	15.10 ± 1.29	40.58 ± 0.99
Model	75.77 ± 3.97^*^	4.13 ± 1.51^**^	15.02 ± 2.01	20.19 ± 0.27^*^
APS	61.66 ± 1.35	17.31 ± 1.17	16.55 ± 1.18	35.42 ± 1.04
YHDH	63.19 ± 1.09	13.47 ± 1.06^*^	18.70 ± 1.23	33.70 ± 1.21
YHDM	65.22 ± 1.83	11.68 ± 0.71^*^	13.53 ± 0.76	27.86 ± 0.70^*^
YHDL	69.08 ± 1.59^*^	13.26 ± 0.93^*^	14.25 ± 0.84	28.49 ± 0.50^*^

Data are presented as the mean ± SD. ^*^ *P <* 0.05. ^**^ *P <* 0.01 vs*.* the control group. Control, mice treated with NS, without TCM; model, mice treated with CTX, without TCM; APS, mice treated with APS, after CTX, was administered; YHDH, YHDM, and YHDL, mice treated with 200, 100 and 50 mg/kg YHD, respectively, after CTX, was administered.

### 3.6 Phagocytosis of peritoneal macrophages

The immunoregulatory effect of YHD was assessed by evaluating the phagocytosis of peritoneal macrophages (Table 3). Compared with the control group, the model group presented decreases in both the phagocytic rate and the phagocytic index (*P* < 0.05, *P* < 0.01), suggesting that CTX treatment impaired the phagocytosis of peritoneal macrophages, which aligns with earlier findings (Marcinkiewicz et al., 1994; Wang et al., 2012). Compared with the model group, YHDH (11.17% ± 0.98%) increased the phagocytic rate (8.17% ± 1.72%) (*P* < 0.05), whereas YHDM and YHDL did not significantly affect the phagocytic rate. All the YHD groups presented an elevated phagocytic index; however, a notable disparity from the control group remained (*P* < 0.05).

**TABLE 3:** Effects of YHD administration for 15 days on the phagocytic rate and index of experimental animals in CTX-induced immunosuppressed mice (n = 10).

Groups	Phagocytic rate (%)	Phagocytic index
Control	12.83 ± 3.37	1.95 ± 0.49
Model	8.17 ± 1.72^*^	1.04 ± 0.07^*^
APS	11.67 ± 1.03	1.47 ± 0.26
YHDH	11.17 ± 0.98	1.13 ± 0.13^*^
YHDM	8.00 ± 1.10^*^	1.14 ± 0.13^*^
YHDL	8.00 ± 1.79^*^	1.25 ± 0.17^*^

Data are expressed as the mean ± SD., Compared with the control group. ^*^ *P <* 0.05. ^**^ *P <* 0.01. Control, mice treated with NS, without TCM; model, mice treated with CTX, without TCM; APS, mice treated with APS, after CTX, was administered; YHDH, YHDM, and YHDL, mice treated with 200, 100 and 50 mg/kg YHD, respectively, after CTX, was administered.

### 3.7 Effects of YHD on cytokines

The data in Table 4 indicate that compared with the control group, the model group presented lower levels of IL-1β and IL-6 (*P* < 0.01, *P* < 0.05). These findings suggest that CTX has the capacity to suppress the innate immune system of animals. Compared with the CTX model group, the groups treated with low, medium, and high doses of YHD had significantly increased serum levels of IL-1β and IL-6 (*P* < 0.01 or *P* < 0.05), indicating that YHD has the potential to enhance innate immunity in CTX-induced immunosuppressed mice. Compared with those in the other groups, the TNF-α levels in the model group were slightly lower. However, after CTX treatment, the serum levels of TNF-α did not significantly differ from those in the control group (*P* > 0.05). In contrast, TNF-α secretion was significantly greater in the APS and YHD groups than in the control group (*P* < 0.05). In the model group, the level of IL-10 significantly increased, surpassing that in the control group (*P* < 0.01). The levels of IL-10 in the APS and YHD groups were notably lower than those in the model group (*P* < 0.01) but still higher than those in the control group. These findings suggest that YHD enhances the immune response by inhibiting the excessive expression of IL-10. The levels of IFN-γ, a crucial immune mediator of both innate and adaptive immunity (Sugisaki et al., 2009; Ding et al., 2022), substantially decreased in response to CTX (*P* < 0.01). Conversely, YHD facilitated the secretion of IFN-γ, thereby mitigating the severity of immunosuppression (Table 4).

**TABLE 4:** Effects of YHD for 15 days on the production of IL-1β, IL-6, TNF-α, IFN-γ and IL-10 in mice with CTX-induced immunosuppression (n = 10).

Groups	IL-1β (pg/mL)	IL-10 (pg/mL)	IL-6 (pg/mL)	TNF-α (pg/mL)	IFN-γ (pg/mL)
Control	73.57 ± 3.59	—	25.80 ± 2.51	8.87 ± 0.31	42.36 ± 0.78
Model	25.73 ± 1.04^**^	222.83 ± 25.08^**^	14.23 ± 2.35^*^	6.97 ± 0.12	11.19 ± 0.95^**^
APS	61.43 ± 2.15^##^	-^##^	20.50 ± 1.65^#^	13.33 ± 0.78^*#^	27.38 ± 0.79^*#^
YHDH	77.10 ± 5.11^*##^	-^##^	35.03 ± 2.72^#^	25.00 ± 3.18^*#^	46.67 ± 1.69^##^
YHDM	47.27 ± 1.47^##^	20.77 ± 9.01^*##^	33.50 ± 4.67^#^	21.60 ± 2.43^*#^	44.76 ± 3.19^##^
YHDL	39.87 ± 9.05^*##^	33.22 ± 1.75^*##^	25.80 ± 2.33^#^	19.22 ± 4.76^*#^	36.62 ± 2.10^#^

“—” indicates that no cytokines were detected; ^*^ *P <* 0.05, ^**^ *P <* 0.01 vs. the control group^; #^ *P <* 0.05, ^##^ *P <* 0.01 vs. the model group. Control, mice treated with NS without TCM; model, mice treated with CTX, without TCM; APS, mice treated with APS, after CTX, was administered; YHDH, YHDM, and YHDL, mice treated with 200, 100 and 50 mg/kg YHD, respectively, after CTX, was administered.

## 4 Discussion

Chemotherapeutics are commonly used to inhibit the proliferation of cancer cells (Chen et al., 2012), with CTX being among the most successful and widely utilized antineoplastic drugs (Emadi et al., 2009). However, CTX causes DNA damage, immune cell depletion, disruption of lymphocyte proliferation and differentiation, and inhibition of humoral and cellular immune responses (Cruz-Chamorro et al., 2007). In the present study, CTX was administered to induce an immunosuppressed state in mice, and the immunomodulatory efficacy of YHD was assessed through *in vivo* experiments.

CTX-treated mice exhibited marked reductions in spleen and thymus index, disrupted histopathology, and impaired immune function. Conversely, YHD treatment significantly increased these organ index and restored structural integrity, suggesting that YHD enhances immune function by promoting the development of immune organs.

The proliferation of splenic lymphocytes in response to mitogens is a well-established method for evaluating lymphocyte immunity (Wang et al., 2016). ConA-induced proliferation reflects T lymphocyte immunity, whereas LPS-induced proliferation reflects B lymphocyte immunity (Wang et al., 2011). Our findings indicate that YHD treatment enhanced the recovery of both T and B lymphocyte proliferation in CTX-treated mice. This effect is likely attributed to the synergistic actions of multiple bioactive compounds in YHD, as partially characterized by our fingerprint analysis (Figure 1). Previous studies have reported that constituents such as sinapine from *S. alba* (Zhou et al., 2022), glycyrrhizin from *G. uralensis* (Zhou et al., 2023), and compounds from *Cervus elaphus* (Zong et al., 2014) can promote splenic lymphocyte proliferation.

Cell cycle analysis revealed that CTX induced splenocyte arrest in the G_0_/G_1_ stage and inhibited DNA synthesis, as evidenced by increased numbers of G_0_/G_1_ cells, decreased numbers of S-phase cells and a decreased proliferation index. YHD treatment reversed these effects by decreasing the number of cells in the G_0_/G_1_ phase and increasing the number of cells in the S phase and the number of cells in the PI, suggesting that YHD primarily mitigates the inhibition of CTX-induced G_0_/G_1_ blockade and DNA synthesis. *Glycyrrhiza uralensis* may contribute to cell cycle regulation, as supported by its presence in formulations such as Shaoyao-Gancao Decoction (Zhou et al., 2023) and Gancao Xiexin Decoction (Yang et al., 2024).

An intriguing observation was the nonmonotonic dose‒response relationship, in which, compared with low (50 mg/kg) and high (200 mg/kg) doses, the medium dose (100 mg/kg) of YHD frequently exhibited superior efficacy. This was evident in multiple parameters, including the spleen index (YHDM: 9.41 ± 0.94 vs. YHDH: 5.19 ± 0.47), body weight recovery (day 26: YHDM 58.5% vs. YHDH 30.4%), and ConA-stimulated lymphocyte proliferation (YHDM: 27.03% ± 0.92% vs. YHDH: 22.73% ± 2.78%). This phenomenon is not uncommon in complex multiherb formulations (Xie et al., 2014) and may be attributed to several factors. First, the inherent complexity of YHD, which contains multiple bioactive compounds from seven medicinal plants, means that at higher concentrations, the synergistic/antagonistic balance among components may shift, leading to a diminished overall effect. Second, this pattern is suggestive of a hormetic or biphasic response, a well-documented biological phenomenon in which a low dose elicits a beneficial effect while a high dose becomes ineffective or even inhibitory (Calabrese, 2014; Sun et al., 2020). In the context of immune modulation, this could be mediated by receptor desensitization or the engagement of compensatory negative feedback loops (e.g., upregulation of regulatory T cells) when the stimulus exceeds an optimal threshold. Third, although no overt toxicity was observed in the high-dose group, the possibility of subtle, subclinical stress or off-target effects cannot be completely excluded (Chatterjee et al., 2011). Importantly, the biological significance of values exceeding control levels should also be considered. In the YHDM group, the spleen index (9.41 ± 0.94) was greater than that in the healthy control group (3.30 ± 0.56). Rather than indicating pathological overactivation, this “overshoot” likely reflects a rebound effect following severe CTX-induced immunosuppression, indicating active immune reconstitution. This interpretation is supported by histopathological analysis (Figures 4, 5), which revealed restored structural integrity without necrosis or inflammatory infiltration. Similar overshoot phenomena have been reported in other immunomodulatory studies, where treated animals temporarily exceeded baseline levels during recovery from immunosuppression (Diwanay et al., 2004). Clinical studies have also revealed that transient immune rebound following chemotherapy, sometimes reaching 150% of pretreatment levels, is associated with favorable clinical outcomes rather than adverse effects, confirming that such overshoot represents physiological restoration rather than pathological compensation (Salem et al., 2012; Braun and Harris, 1981; Harris et al., 1976).

Macrophages play a critical role in nonspecific immunity by ingesting and eliminating foreign antigens (Wang et al., 2020). The phagocytic activity of peritoneal macrophages, a marker of nonspecific immunity (Chen et al., 2010), was markedly increased by YHD, indicating an enhanced nonspecific immune response in CTX-treated mice.

Cytokines are essential for immune cell communication, development, and differentiation (Liu et al., 2019). CTX decreased the levels of the proinflammatory cytokines IL-1β, IL-6, and TNF-α, whereas YHD increased them. IL-6 regulates B and T-cell proliferation and differentiation (Ren et al., 2014), and TNF-α is critical for inflammation and host defense (Idriss and Naismith, 2000). These findings indicate that YHD modulates innate immunity through cytokine regulation. Conversely, the expression of IL-10, an anti-inflammatory cytokine that maintains immune homeostasis (Bedke et al., 2019), was significantly elevated by CTX but normalized by YHD administration, suggesting that YHD helps restore immune equilibrium.

Compared with that in the CTX group, the expression of IFN-γ, a key regulator of cellular immunity with broad-spectrum antiviral properties (Raymond and Wilkie, 2004), was notably elevated in the YHD-treated group, indicating that YHD may mitigate immunosuppression by increasing IFN-γ secretion. These effects are likely mediated by the seven herbs in YHD. For instance, *S. alba* increases the release of IL-1β and TNF-α (Guo et al., 2013), whereas *Z. officinale* regulates cytokine balance (Ju et al., 2021). Acteoside and catalpol modulate cytokine levels (Wu et al., 2022; Zhang et al., 2019), and *Cinnamomum cassia* volatile oils promote the release of IL-1β and IL-6 (Ni et al., 2016). *Rehmannia glutinosa* has also been reported to modulate inflammatory cytokine production; for instance, its components have been shown to inhibit the production of IL-6 and other proinflammatory mediators in LPS-stimulated RAW264.7 macrophages (Liu et al., 2012). These findings suggest that TCMs can regulate cytokine production in cells under various conditions rather than simply increasing or decreasing cytokine levels.

Compared with the effects in the model group, low-dose YHD had the strongest effect on LPS-stimulated B-cell proliferation (12.42% ± 4.34% vs. −6.33% ± 0.85%), whereas medium- and high-dose YHD had weaker effects (YHDM: 7.58% ± 4.27%; YHDH: 6.60% ± 2.61%). This inverse dose‒response may reflect differential effects on lymphocyte subsets, where lower concentrations selectively promote B-cell activity, whereas higher doses engage regulatory mechanisms. Similar biphasic effects have been reported for other botanical extracts (Chatterjee et al., 2011; Guo et al., 2013), highlighting the complexity of multicomponent formulations.

### 4.1 Interpretation of cytokine modulation in the context of immunosuppression

While excessive proinflammatory mediators are associated with pathological inflammation, it is crucial to interpret our findings within the context of CTX-induced immunosuppression. In this model, baseline cytokine levels are pathologically low (Farooqui et al., 2026; Ilyas et al., 2025). The increases observed after YHD treatment represent a restorative effect, reconstituting immune competence. Importantly, as shown in Table 4, the cytokine levels in the optimal YHD groups approached, but did not exceed, those in the healthy control group. This interpretation is supported by histopathological analysis (Figures 4, 5), which revealed restored structural integrity without necrosis or pathological inflammatory infiltration (Calabrese et al., 2018; Ping et al., 2020).

### 4.2 Chemical characterization and quality control

While we established an HPLC fingerprint of YHD and confirmed key compounds by reference standards (Figure 1), this analysis was primarily qualitative. We did not perform absolute quantification of the bioactive constituents. Recent studies have established HPLC fingerprinting methods and quantified multiple indicator components for YHD, demonstrating the feasibility of comprehensive quality control (Zekang et al., 2023; Zh et al., 2023). Future studies should employ advanced techniques such as HPLC‒MS‒MS or UPLC‒Q‒TOF‒MS‒MS for the simultaneous quantification of major bioactive markers (Sijia et al., 2025; Cai et al., 2025), which would ensure batch-to-batch consistency and enable pharmacokinetic and dose‒response studies.

### 4.3 Limitations of the WST-8 assay for proliferation assessment

Several limitations should be acknowledged. First, the WST-8 assay measures cellular metabolic activity as a surrogate for viable cell number rather than directly quantifying cell division (Ishiyama et al., 1996) and may be influenced by metabolic changes independent of proliferation (Wang et al., 2010). To address this, we performed cell cycle analysis by flow cytometry (Table 2; Figure 7) as a more direct functional assay. The significant increase in S-phase cells in the YHD-treated groups provides stronger evidence for enhanced proliferation (Pozarowski et al., 2004), and the consistency between the WST-8 and cell cycle data reinforces our conclusions. However, more definitive methods, such as CFSE dilution, BrdU/EdU incorporation, or Ki67 staining (Pozarowski et al., 2004; Salic and Mitchison, 2008; Quah and Parish, 2010), were not performed. Future studies should incorporate these complementary methods to validate and extend our findings (Gratzner, 1982).

### 4.4 Proposed mechanisms and future directions

While our study provides robust evidence that YHD protects against CTX-induced immunosuppression, the precise underlying molecular mechanisms remain to be fully elucidated. On the basis of our findings and the literature, we propose several testable hypotheses:

Cell cycle modulation. YHD increased the proportion of splenic lymphocytes in the S and G_2_/M phases, suggesting that YHD facilitates DNA repair and cell cycle checkpoint progression. Natural products, including γ-H2AX, p53, p21, and checkpoint kinases (ATM/ATR, Chk1), can modulate key DNA damage response pathways (Liang et al., 2025; Pargi et al., 2022).

Antioxidant Potential. Constituents such as catalpol, glycyrrhizin, and gingerols possess antioxidant properties (Ju et al., 2021; Wu et al., 2022; Zhang et al., 2019). CTX induces immunosuppression partly through ROS-mediated oxidative DNA damage. YHD may exert cytoprotective effects by enhancing endogenous antioxidant defences or activating the Nrf2/HO-1 pathway (You et al., 2019).

Cytokine-mediated survival. Restoration of IL-2 and IL-6 may activate pro-survival pathways such as the PI3K/Akt pathway, increasing the resistance of lymphocytes to apoptosis (Diwanay et al., 2004).

We acknowledge that direct validation of these hypotheses, including the assessment of DNA damage markers (γ-H2AX and 8-OHdG), antioxidant enzyme activities, and signaling pathways (p53, Nrf2, PI3K/Akt, and ATR/Chk1), was beyond the scope of this efficacy-focused study (Jackson and Bartek, 2009; Liu et al., 2017). Future investigations employing these molecular endpoints are warranted.

### 4.5 Limitations of the nontumor model and future directions

A critical limitation is the absence of a tumor-bearing model. CTX is primarily used in cancer treatment, and the ultimate goal of YHD is as an adjuvant therapy for chemotherapy patients (Emadi et al., 2009). Evaluating YHD in tumor-bearing models is essential for determining whether the observed immunoprotection inadvertently protects tumors (interfering with CTX efficacy) or enhances antitumor immunity while protecting the host (Tomar et al., 2025).

The primary objective of this study was to investigate the direct effects of YHD on the immune system in a controlled, nontumor setting—a necessary foundational step before the development of more complex tumor-bearing models (Diwanay et al., 2004). Encouragingly, studies have begun exploring YHD in tumor-bearing models. Xiang et al. (2006) reported antitumor effects in tumor-bearing mice, whereas Jia (2016) reported that YHD inhibited tumor growth in S180 sarcoma-bearing mice, improved immune organ index, and downregulated VEGF/HIF-1α expression, suggesting antiangiogenic effects. These studies indicate that YHD may have dual benefits: antitumor activity and immune protection.

On the basis of our findings and the literature, future research directions include evaluating syngeneic tumor models to investigate YHD combined with CTX in Lewis lung carcinoma or B16 melanoma models (Yuan et al., 2023; Ahmad et al., 2012), with key endpoints encompassing antitumor efficacy, immunoprotective effects, and the tumor immune microenvironment. In parallel, assessment of interference with CTX activity is warranted to compare the antitumor efficacy of CTX alone with that of CTX plus YHD, thereby determining whether the observed immunoprotection compromises chemotherapy (Diwanay et al., 2004). Furthermore, mechanistic studies should explore the involvement of TLR4/NF-κB signaling, angiogenesis (You et al., 2019), and cytotoxic immune cell function. Finally, dose optimization studies are needed to determine whether the optimal immunoprotective dose identified in healthy mice (100 mg/kg) translates effectively to tumor-bearing conditions.

Addressing these questions is crucial for the clinical translation of YHD as an adjunctive therapy for chemotherapy patients. The results of the present study provide the essential pharmacological foundation for these future investigations.

## 5 Conclusion

The findings of this study suggest that YHD can regulate immune organ status, enhance macrophage phagocytic function, modulate blood cytokine levels, and improve splenic lymphocyte proliferation in immunosuppressed mice, which implies that YHD has potential for the treatment of immunosuppressed patients receiving chemotherapy and as a supplementary treatment for conditions characterized by immune deficiency.

## Data Availability

The raw data supporting the conclusions of this article will be made available by the authors, without undue reservation.
