# Supplementary material for: Immunomodulatory effects of Yang He decoction on cyclophosphamide-induced immunosuppression in mice: restoration of immune organ integrity and cytokine balance
Source: Front Pharmacol. 2026 Jun 16;17:1805534. doi: 10.3389/fphar.2026.1805534 (PMC13315013; doi:10.3389/fphar.2026.1805534)
Supplement: Supplementary file 1 [file Table1.docx]

# Table S1. Main composition of YHD

| Botanical plant name | Pharmaceutical name | Chinese name | Family | Part used | Weight (g) | Origin (Province, PR China) | Obtain time | Voucher no. |
| --- | --- | --- | --- | --- | --- | --- | --- | --- |
| *Rehmannia glutinosa* (Gaertn.) DC. | Rehmanniae Radix Praeparata | Shu Di Huang | Orobanchaceae | Radix | 30 | Henan | Oct. 2017 | 2112YHD01 |
| *Neolitsea cassia* (L.) Kosterm. | Cinnamomi Cortex | Rou Gui | Lauraceae | Cortex | 3 | Guangxi Province | Sept. 2019 | 2112YHD02 |
| *Ephedra sinica* Stapf | Ephedrae Herba | Ma Huang | Ephedraceae | Herba | 2 | Neimenggu Province | Oct. 2017 | 2112YHD03 |
| *Cervus elaphus* Linnaeus | Cervi Cornu | Lu Jiao Jiao | Cervidae | Cornu | 9 | Jilin Province | Mar. 2020 | 2112YHD04 |
| *Sinapis alba* L. | Sinapis Semen | Jie Zi | Brassicaceae | Semen | 6 | Hebei Province | Aug. 2018 | 2112YHD05 |
| *Zingiber officinale* Roscoe | Zingiber offcinale Rose | Jiang Tan | Zingiberaceae | Rhizome | 2 | Yunnan Province | Dec. 2019 | 2112YHD06 |
| *Glycyrrhiza uralensis* Fisch. ex DC. | Glycyrrhizae Radix et Rhizoma | Gan Cao | Fabaceae | Rhizome | 3 | Gansu Province | Oct. 2020 | 2112YHD07 |
