# Supplementary material for: Immunomodulatory effects of Yang He decoction on cyclophosphamide-induced immunosuppression in mice: restoration of immune organ integrity and cytokine balance
Source: Front Pharmacol. 2026 Jun 16;17:1805534. doi: 10.3389/fphar.2026.1805534 (PMC13315013; doi:10.3389/fphar.2026.1805534)
Supplement: Supplementary file 2 [file Table2.doc]

**Table S2 Raw body weight data of mice**

# Table S2-1. Raw body weight data of control mice during days 1-12****(g)****

| No. | d1 | d2 | d3 | d7 | d11 | d12 |
| --- | --- | --- | --- | --- | --- | --- |
| 1 | 20.00 | 21.04 | 22.40 | 26.60 | 30.56 | 30.60 |
| 2 | 22.00 | 23.21 | 25.70 | 30.52 | 31.60 | 31.87 |
| 3 | 18.00 | 19.44 | 21.10 | 24.52 | 27.42 | 27.80 |
| 4 | 20.00 | 20.90 | 22.66 | 27.32 | 30.82 | 30.88 |
| 5 | 18.00 | 19.22 | 20.38 | 24.48 | 27.34 | 27.58 |
| 6 | 19.00 | 20.69 | 21.55 | 25.69 | 27.46 | 27.59 |
| 7 | 20.20 | 20.92 | 23.51 | 27.23 | 30.59 | 30.30 |
| 8 | 20.20 | 21.05 | 22.66 | 27.15 | 29.29 | 29.50 |
| 9 | 21.00 | 21.99 | 24.47 | 28.54 | 31.71 | 31.88 |
| 10 | 22.00 | 23.12 | 24.67 | 29.40 | 32.00 | 32.30 |
| 11 | 20.00 | 21.10 | 23.28 | 26.68 | 30.10 | 30.08 |
| 12 | 22.00 | 23.29 | 24.68 | 29.29 | 32.10 | 32.43 |
| 13 | 21.00 | 22.31 | 24.70 | 27.15 | 31.51 | 31.76 |
| 14 | 20.00 | 21.35 | 22.47 | 27.68 | 29.28 | 29.64 |
| 15 | 18.00 | 19.29 | 21.01 | 23.73 | 26.93 | 26.96 |
| 16 | 18.20 | 19.57 | 20.44 | 23.85 | 26.72 | 26.76 |
| 17 | 18.80 | 20.29 | 21.85 | 24.61 | 26.16 | 26.85 |
| 18 | 22.00 | 23.83 | 25.01 | 28.76 | 32.41 | 32.74 |
| 19 | 22.00 | 23.94 | 24.93 | 29.33 | 32.71 | 32.86 |
| 20 | 18.20 | 19.92 | 20.39 | 23.85 | 26.89 | 27.00 |
| Average | 20.03 | 21.32 | 22.89 | 26.82 | 29.68 | 29.87 |
| SD | 1.50 | 1.53 | 1.75 | 2.12 | 2.23 | 2.22 |

# Table S2-2. Raw body weight data of model mice during days 1-12****(g)****

| No. | d1 | d2 | d3 | d7 | d11 | d12 |
| --- | --- | --- | --- | --- | --- | --- |
| 1 | 20 | 20.32 | 19.76 | 19.86 | 20.84 | 20.89 |
| 2 | 22 | 22.35 | 21.76 | 21.87 | 22.99 | 23.02 |
| 3 | 18 | 18.25 | 17.86 | 17.95 | 18.89 | 18.92 |
| 4 | 20 | 20.28 | 19.74 | 19.84 | 20.9 | 20.97 |
| 5 | 18 | 18.29 | 17.71 | 17.87 | 18.82 | 18.89 |
| 6 | 19 | 19.27 | 18.81 | 18.91 | 19.82 | 19.88 |
| 7 | 20.2 | 20.48 | 19.88 | 20.01 | 21 | 21.08 |
| 8 | 20.2 | 20.52 | 20.3 | 20.18 | 21.23 | 21.28 |
| 9 | 21 | 21.33 | 20.64 | 20.87 | 21.81 | 21.9 |
| 10 | 22 | 22.4 | 22.24 | 22.14 | 23.23 | 23.27 |
| 11 | 20 | 20.31 | 19.76 | 19.86 | 20.76 | 20.82 |
| 12 | 22 | 22.31 | 21.71 | 21.83 | 22.96 | 22.99 |
| 13 | 21 | 21.32 | 20.98 | 21.09 | 22.12 | 22.13 |
| 14 | 20 | 20.28 | 19.76 | 19.85 | 20.87 | 20.86 |
| 15 | 18 | 18.23 | 17.94 | 18.06 | 18.98 | 18.94 |
| 16 | 18.2 | 18.47 | 18.14 | 18.27 | 19.17 | 19.21 |
| 17 | 18.8 | 19.08 | 18.78 | 18.85 | 19.84 | 19.83 |
| 18 | 22 | 22.32 | 21.69 | 21.95 | 22.99 | 22.99 |
| 19 | 22 | 22.32 | 21.78 | 21.91 | 23.03 | 23.09 |
| 20 | 18.2 | 18.49 | 18.12 | 18.23 | 19.15 | 19.22 |
| 21 | 18.3 | 18.56 | 17.95 | 18.37 | 19.29 | 19.33 |
| 22 | 19.6 | 19.87 | 19.36 | 19.48 | 20.44 | 20.54 |
| 23 | 20.5 | 20.81 | 20.17 | 20.27 | 21.14 | 21.22 |
| 24 | 21.2 | 21.52 | 20.99 | 21.01 | 22.03 | 22.09 |
| 25 | 20 | 20.35 | 19.82 | 19.88 | 20.82 | 20.87 |
| 26 | 18.4 | 18.58 | 18.05 | 18.14 | 19.07 | 19.1 |
| 27 | 21.6 | 21.95 | 21.32 | 21.4 | 22.57 | 22.55 |
| 28 | 19.5 | 19.75 | 19.31 | 19.39 | 20.38 | 20.41 |
| 29 | 18.4 | 18.7 | 18.44 | 18.54 | 19.47 | 19.5 |
| 30 | 19.6 | 19.93 | 19.56 | 19.67 | 20.68 | 20.73 |
| 31 | 18.4 | 18.69 | 18.16 | 18.26 | 19.16 | 19.17 |
| 32 | 21.6 | 21.91 | 21.36 | 21.46 | 22.46 | 22.47 |
| 33 | 21.8 | 22.15 | 21.91 | 21.99 | 23.31 | 23.32 |
| 34 | 20.4 | 20.71 | 20.33 | 20.35 | 21.35 | 21.4 |
| 35 | 20.4 | 20.71 | 20.44 | 20.56 | 21.69 | 21.72 |
| 36 | 19.9 | 20.16 | 19.66 | 19.74 | 20.7 | 20.68 |
| 37 | 18.9 | 19.13 | 18.56 | 18.65 | 19.6 | 19.67 |
| 38 | 21.6 | 21.93 | 21.43 | 21.47 | 22.52 | 22.59 |
| 39 | 20.9 | 21.28 | 20.97 | 21.06 | 21.99 | 22.12 |
| 40 | 19.1 | 19.41 | 19.16 | 19.23 | 20.22 | 20.24 |
| 41 | 20.4 | 20.67 | 20.11 | 20.21 | 21.23 | 21.29 |
| 42 | 21.3 | 21.6 | 20.98 | 21.08 | 22.03 | 22.33 |
| 43 | 18.6 | 18.92 | 18.62 | 18.7 | 19.6 | 19.6 |
| 44 | 19.5 | 19.78 | 19.61 | 19.7 | 20.54 | 20.56 |
| 45 | 20.4 | 20.67 | 20.05 | 20.2 | 21.26 | 21.27 |
| 46 | 21.3 | 21.64 | 21.19 | 21.33 | 22.44 | 22.4 |
| 47 | 20.5 | 20.74 | 20.13 | 20.24 | 21.1 | 21.17 |
| 48 | 19.6 | 19.84 | 19.31 | 19.4 | 20.24 | 20.24 |
| 49 | 18.4 | 18.73 | 18.44 | 18.46 | 19.56 | 19.58 |
| 50 | 19.3 | 19.61 | 19.23 | 19.33 | 20.28 | 20.43 |
| 51 | 18.6 | 18.86 | 18.37 | 18.48 | 19.38 | 19.38 |
| 52 | 20.4 | 20.75 | 20.23 | 20.37 | 21.41 | 21.44 |
| 53 | 21.8 | 22.15 | 21.58 | 21.71 | 22.81 | 22.82 |
| 54 | 21 | 21.32 | 20.75 | 20.89 | 22.1 | 22.12 |
| 55 | 19.8 | 20.02 | 19.46 | 19.57 | 20.66 | 20.73 |
| 56 | 18.6 | 18.87 | 18.53 | 18.63 | 19.58 | 19.58 |
| 57 | 19.8 | 20.16 | 19.64 | 19.73 | 20.78 | 20.83 |
| 58 | 20.3 | 20.59 | 20.33 | 20.4 | 21.37 | 21.35 |
| 59 | 21.2 | 21.54 | 20.99 | 21.09 | 22.17 | 22.23 |
| 60 | 19.6 | 19.95 | 19.56 | 19.64 | 20.63 | 20.65 |
| 61 | 18.3 | 18.59 | 18.19 | 18.28 | 19.19 | 19.24 |
| 62 | 19.2 | 19.45 | 18.87 | 18.91 | 19.91 | 19.94 |
| 63 | 21.3 | 21.56 | 20.92 | 21.03 | 22.08 | 22.03 |
| 64 | 20.6 | 20.9 | 20.65 | 20.72 | 21.74 | 21.82 |
| 65 | 18.9 | 19.22 | 18.82 | 18.94 | 19.88 | 19.93 |
| 66 | 19.4 | 19.65 | 19.13 | 19.25 | 20.19 | 20.25 |
| 67 | 19.9 | 20.22 | 19.66 | 19.78 | 20.72 | 20.74 |
| 68 | 20.6 | 20.91 | 20.53 | 20.65 | 21.65 | 21.66 |
| 69 | 21.5 | 21.8 | 21.43 | 21.52 | 22.69 | 22.79 |
| 70 | 21.1 | 21.41 | 20.83 | 20.98 | 22.21 | 22.22 |
| 71 | 18.4 | 18.69 | 18.25 | 18.35 | 19.2 | 19.29 |
| 72 | 18.1 | 18.35 | 17.88 | 17.95 | 19.03 | 19.14 |
| 73 | 19.3 | 19.63 | 19.27 | 19.38 | 20.28 | 20.36 |
| 74 | 18.4 | 18.7 | 18.33 | 18.43 | 19.33 | 19.34 |
| 75 | 19.4 | 19.73 | 19.28 | 19.36 | 20.19 | 20.2 |
| 76 | 20.6 | 20.93 | 20.53 | 20.63 | 21.69 | 21.74 |
| 77 | 21 | 21.34 | 20.75 | 20.87 | 21.89 | 21.89 |
| 78 | 20.9 | 21.18 | 20.58 | 20.7 | 21.8 | 21.82 |
| 79 | 21.3 | 21.57 | 21.19 | 21.28 | 22.2 | 22.25 |
| 80 | 22 | 22.37 | 21.91 | 21.98 | 23.16 | 23.19 |
| 81 | 18.8 | 19.03 | 18.54 | 18.64 | 19.58 | 19.59 |
| 84 | 19.8 | 20.02 | 19.46 | 19.59 | 20.62 | 20.65 |
| 85 | 18.9 | 19.16 | 18.56 | 18.71 | 19.67 | 19.69 |
| 86 | 20.6 | 20.97 | 20.62 | 20.66 | 21.69 | 21.71 |
| 87 | 21.2 | 21.41 | 20.8 | 20.93 | 21.88 | 21.88 |
| 88 | 20.3 | 20.62 | 20.34 | 20.43 | 21.25 | 21.3 |
| 89 | 18.7 | 18.98 | 18.61 | 18.74 | 19.7 | 19.68 |
| 90 | 19.6 | 19.85 | 19.33 | 19.54 | 20.44 | 20.52 |
| 91 | 20.5 | 20.87 | 20.44 | 20.52 | 21.56 | 21.66 |
| 92 | 21.4 | 21.72 | 21.14 | 21.29 | 22.32 | 22.42 |
| 93 | 18.9 | 19.2 | 18.65 | 18.77 | 19.82 | 19.89 |
| 94 | 19.6 | 19.95 | 19.54 | 19.55 | 21.16 | 21.17 |
| 95 | 18.9 | 19.16 | 18.64 | 18.71 | 19.68 | 19.68 |
| 96 | 19.4 | 19.75 | 19.32 | 19.35 | 20.17 | 20.23 |
| 97 | 18.9 | 19.17 | 19.01 | 19.09 | 19.77 | 19.77 |
| 98 | 19.4 | 19.67 | 19.17 | 19.34 | 20.24 | 20.34 |
| 99 | 20.4 | 20.75 | 20.27 | 20.39 | 21.44 | 21.49 |
| 100 | 21.5 | 21.89 | 21.61 | 21.55 | 22.63 | 22.67 |
| Average | 19.8 | 18.98 | 19.82 | 19.92 | 20.91 | 20.95 |
| SD | 2.16 | 5.09 | 1.16 | 1.15 | 1.21 | 1.22 |

# Table S2-3. Body weight data of control mice during days 13-26****(g)****

| No. | d13 | d14 | d15 | d16 | d17 | d18 | d19 | d20 | d21 | d22 | d23 | d24 | d25 | d26 |
| --- | --- | --- | --- | --- | --- | --- | --- | --- | --- | --- | --- | --- | --- | --- |
| 1 | 31.60 | 32.40 | 33.00 | 34.20 | 35.00 | 36.80 | 37.60 | 38.60 | 39.60 | 40.00 | 41.00 | 42.00 | 43.20 | 44.00 |
| 2 | 34.14 | 35.90 | 36.48 | 37.93 | 38.92 | 40.90 | 41.71 | 42.88 | 43.89 | 44.22 | 45.36 | 46.53 | 48.16 | 50.71 |
| 3 | 28.75 | 29.21 | 30.01 | 31.28 | 32.00 | 33.21 | 33.97 | 34.85 | 35.57 | 36.61 | 37.57 | 38.41 | 39.85 | 41.85 |
| 4 | 31.36 | 31.78 | 33.78 | 35.18 | 35.88 | 37.38 | 37.94 | 38.50 | 39.68 | 40.48 | 41.18 | 41.88 | 43.16 | 44.92 |
| 5 | 28.35 | 29.11 | 30.13 | 30.98 | 31.54 | 33.12 | 34.06 | 35.33 | 35.50 | 36.00 | 36.61 | 36.97 | 38.05 | 40.05 |
| 6 | 29.28 | 30.23 | 31.33 | 32.13 | 32.59 | 34.28 | 35.23 | 36.12 | 36.39 | 37.13 | 38.00 | 38.46 | 39.43 | 40.93 |
| 7 | 32.16 | 33.11 | 33.63 | 34.64 | 35.07 | 36.87 | 37.51 | 38.68 | 39.47 | 40.34 | 41.35 | 42.20 | 42.95 | 44.40 |
| 8 | 30.26 | 31.25 | 33.79 | 34.87 | 35.53 | 37.29 | 38.24 | 39.09 | 40.30 | 41.21 | 42.14 | 43.13 | 44.28 | 45.25 |
| 9 | 32.05 | 32.95 | 35.53 | 37.70 | 38.05 | 39.59 | 40.40 | 41.12 | 41.71 | 42.53 | 43.20 | 44.39 | 45.23 | 47.17 |
| 10 | 32.54 | 33.59 | 36.06 | 37.36 | 37.97 | 39.91 | 41.51 | 42.33 | 43.23 | 43.89 | 45.43 | 46.07 | 46.24 | 47.08 |
| 11 | 31.08 | 31.96 | 33.08 | 34.04 | 35.36 | 37.70 | 38.84 | 39.92 | 41.14 | 41.48 | 42.68 | 43.70 | 45.22 | 46.30 |
| 12 | 32.96 | 34.80 | 35.77 | 37.16 | 37.95 | 40.79 | 41.47 | 42.57 | 43.69 | 44.95 | 45.89 | 46.71 | 48.29 | 49.19 |
| 13 | 32.32 | 33.92 | 35.39 | 36.25 | 37.04 | 39.10 | 39.88 | 41.31 | 41.85 | 43.05 | 44.60 | 45.40 | 46.49 | 48.24 |
| 14 | 30.54 | 32.54 | 33.94 | 34.42 | 35.38 | 37.08 | 38.24 | 39.52 | 39.86 | 40.40 | 41.46 | 42.48 | 42.60 | 43.64 |
| 15 | 28.06 | 28.37 | 29.75 | 30.64 | 31.34 | 32.90 | 34.11 | 34.63 | 35.17 | 35.93 | 36.86 | 37.40 | 38.83 | 39.53 |
| 16 | 27.59 | 28.32 | 29.56 | 30.81 | 31.45 | 33.01 | 33.91 | 34.87 | 35.25 | 35.95 | 36.45 | 36.98 | 38.11 | 39.95 |
| 17 | 28.75 | 29.48 | 30.34 | 31.58 | 32.45 | 34.01 | 34.86 | 35.85 | 36.32 | 37.00 | 37.49 | 37.68 | 38.58 | 40.91 |
| 18 | 34.03 | 35.13 | 36.04 | 37.00 | 38.19 | 40.13 | 41.07 | 42.31 | 43.05 | 43.69 | 44.40 | 44.99 | 46.02 | 47.70 |
| 19 | 33.79 | 35.13 | 36.19 | 37.16 | 38.52 | 41.07 | 41.65 | 42.86 | 43.96 | 44.88 | 45.28 | 45.50 | 47.12 | 48.73 |
| 20 | 28.19 | 29.25 | 30.61 | 31.18 | 32.18 | 34.07 | 35.07 | 36.13 | 36.76 | 37.24 | 37.44 | 37.98 | 38.93 | 40.82 |
| Average | 30.89 | 31.92 | 33.22 | 34.32 | 35.12 | 36.96 | 37.86 | 38.87 | 39.62 | 40.35 | 41.22 | 41.94 | 43.04 | 44.57 |
| SD | 2.13 | 2.43 | 2.48 | 2.62 | 2.69 | 2.93 | 2.91 | 2.98 | 3.18 | 3.23 | 3.38 | 3.51 | 3.55 | 3.50 |

# Table S2-4. Body weight data of APS-treated mice during days 13-26****(g)****

| No. | d13 | d14 | d15 | d16 | d17 | d18 | d19 | d20 | d21 | d22 | d23 | d24 | d25 | d26 |
| --- | --- | --- | --- | --- | --- | --- | --- | --- | --- | --- | --- | --- | --- | --- |
| 1 | 18.50 | 19.55 | 18.75 | 21.09 | 20.63 | 20.32 | 20.23 | 21.05 | 21.47 | 22.11 | 22.71 | 22.64 | 22.44 | 23.31 |
| 2 | 20.37 | 21.61 | 20.55 | 23.07 | 22.56 | 22.28 | 22.16 | 23.08 | 23.57 | 24.26 | 25.01 | 24.95 | 24.72 | 25.64 |
| 3 | 16.78 | 17.80 | 17.06 | 19.28 | 18.94 | 18.74 | 18.64 | 19.41 | 19.68 | 20.36 | 21.10 | 21.18 | 20.85 | 21.79 |
| 4 | 18.57 | 19.70 | 19.10 | 21.45 | 21.07 | 20.85 | 20.72 | 21.52 | 21.83 | 22.62 | 23.46 | 23.38 | 23.16 | 24.02 |
| 5 | 16.70 | 17.68 | 17.05 | 19.08 | 18.74 | 18.43 | 18.31 | 19.06 | 19.26 | 20.19 | 20.70 | 20.62 | 20.44 | 21.03 |
| 6 | 17.63 | 18.64 | 17.98 | 20.14 | 19.68 | 19.35 | 19.25 | 20.06 | 20.83 | 21.09 | 21.35 | 21.28 | 21.07 | 22.01 |
| 7 | 18.70 | 19.85 | 19.13 | 21.50 | 21.03 | 20.64 | 20.55 | 21.42 | 21.40 | 22.01 | 22.29 | 22.23 | 22.17 | 23.16 |
| 8 | 18.95 | 20.18 | 19.33 | 21.65 | 21.26 | 21.03 | 20.98 | 21.81 | 21.93 | 22.49 | 23.08 | 23.07 | 22.87 | 23.92 |
| 9 | 19.46 | 20.69 | 19.81 | 22.08 | 21.63 | 21.29 | 21.13 | 21.81 | 22.33 | 22.97 | 24.20 | 24.16 | 23.69 | 24.47 |
| 10 | 20.50 | 21.67 | 20.81 | 23.35 | 22.93 | 22.56 | 22.48 | 23.31 | 23.58 | 24.39 | 25.38 | 25.37 | 25.30 | 26.47 |
| 11 | 18.48 | 19.53 | 18.69 | 21.07 | 20.62 | 20.27 | 20.27 | 21.09 | 21.45 | 22.13 | 22.86 | 22.87 | 22.71 | 23.28 |
| 12 | 20.34 | 21.54 | 20.63 | 23.14 | 22.66 | 22.28 | 22.27 | 23.18 | 23.62 | 24.28 | 25.35 | 25.45 | 25.19 | 26.09 |
| 13 | 19.62 | 20.72 | 19.96 | 22.50 | 22.11 | 21.89 | 21.77 | 22.61 | 22.88 | 23.84 | 24.61 | 24.57 | 24.35 | 25.27 |
| 14 | 18.48 | 19.55 | 18.69 | 20.99 | 20.59 | 20.27 | 20.12 | 20.91 | 21.58 | 22.25 | 22.80 | 22.61 | 22.58 | 23.37 |
| 15 | 16.82 | 17.78 | 17.07 | 19.18 | 18.74 | 18.42 | 18.30 | 19.01 | 19.46 | 20.09 | 20.26 | 20.22 | 20.26 | 20.80 |
| 16 | 17.01 | 18.03 | 17.21 | 19.36 | 18.99 | 18.63 | 18.46 | 19.36 | 19.59 | 20.19 | 20.76 | 20.80 | 20.67 | 21.37 |
| 17 | 17.63 | 18.70 | 17.75 | 19.98 | 19.60 | 19.28 | 19.19 | 20.18 | 20.50 | 20.91 | 21.43 | 21.35 | 21.08 | 21.80 |
| 18 | 20.46 | 21.68 | 20.95 | 23.66 | 23.18 | 22.90 | 22.90 | 23.79 | 24.11 | 24.98 | 25.64 | 25.65 | 24.76 | 25.60 |
| 19 | 20.31 | 21.66 | 20.91 | 23.37 | 22.93 | 22.51 | 22.34 | 23.35 | 23.73 | 24.56 | 25.19 | 24.98 | 24.79 | 25.42 |
| 20 | 16.94 | 17.89 | 17.18 | 19.36 | 19.00 | 18.71 | 18.53 | 19.38 | 19.71 | 20.36 | 20.68 | 20.81 | 20.67 | 21.24 |
| Average | 18.61 | 19.72 | 18.93 | 21.26 | 20.84 | 20.53 | 20.43 | 21.27 | 21.63 | 22.30 | 22.94 | 22.91 | 22.69 | 23.50 |
| SD | 1.36 | 1.46 | 1.42 | 1.57 | 1.54 | 1.53 | 1.55 | 1.59 | 1.59 | 1.66 | 1.83 | 1.82 | 1.75 | 1.83 |

# Table S2-5. Body weight data of YHDL-treated mice during days 13-26****(g)****

| No. | d13 | d14 | d15 | d16 | d17 | d18 | d19 | d20 | d21 | d22 | d23 | d24 | d25 | d26 |
| --- | --- | --- | --- | --- | --- | --- | --- | --- | --- | --- | --- | --- | --- | --- |
| 1 | 17.49 | 18.33 | 18.03 | 18.49 | 19.84 | 19.82 | 19.74 | 20.38 | 22.44 | 21.88 | 23.67 | 23.22 | 22.59 | 22.79 |
| 2 | 18.55 | 19.60 | 19.24 | 19.92 | 21.38 | 21.27 | 21.19 | 22.06 | 24.23 | 23.64 | 25.37 | 24.81 | 24.48 | 24.76 |
| 3 | 19.02 | 20.02 | 19.71 | 20.28 | 21.84 | 21.78 | 21.69 | 22.60 | 24.63 | 24.69 | 26.96 | 26.31 | 25.76 | 25.75 |
| 4 | 19.99 | 21.06 | 20.42 | 21.00 | 22.57 | 22.54 | 22.39 | 23.41 | 25.48 | 25.89 | 28.36 | 26.82 | 25.99 | 26.40 |
| 5 | 18.93 | 19.93 | 19.79 | 20.30 | 21.58 | 21.47 | 21.17 | 22.00 | 23.91 | 24.05 | 25.98 | 25.13 | 24.27 | 24.72 |
| 6 | 17.11 | 17.97 | 17.47 | 18.05 | 19.54 | 19.40 | 19.44 | 20.12 | 22.27 | 21.63 | 23.28 | 22.74 | 22.00 | 22.57 |
| 7 | 20.30 | 21.46 | 20.88 | 21.38 | 22.98 | 22.56 | 22.67 | 23.64 | 26.43 | 26.04 | 28.42 | 28.00 | 27.07 | 27.78 |
| 8 | 18.54 | 19.52 | 19.25 | 19.74 | 21.16 | 21.30 | 21.19 | 21.76 | 24.10 | 23.64 | 25.80 | 25.24 | 24.66 | 25.27 |
| 9 | 17.68 | 18.63 | 18.37 | 18.77 | 20.15 | 20.16 | 20.13 | 21.01 | 23.31 | 22.36 | 24.01 | 23.49 | 22.80 | 23.08 |
| 10 | 18.88 | 19.90 | 19.55 | 20.08 | 21.48 | 21.43 | 21.41 | 22.39 | 24.63 | 24.15 | 26.59 | 26.18 | 25.73 | 26.18 |
| 11 | 17.32 | 18.38 | 18.03 | 18.45 | 19.79 | 19.79 | 19.70 | 20.42 | 22.57 | 22.13 | 23.89 | 23.67 | 23.29 | 23.21 |
| 12 | 20.24 | 21.39 | 21.01 | 21.71 | 23.26 | 23.21 | 23.18 | 23.90 | 26.03 | 25.73 | 27.55 | 27.08 | 26.37 | 26.27 |
| 13 | 21.11 | 22.30 | 21.83 | 22.31 | 23.95 | 23.89 | 23.78 | 24.63 | 28.29 | 27.31 | 29.79 | 29.05 | 28.37 | 28.92 |
| 14 | 19.33 | 20.35 | 19.98 | 20.49 | 22.07 | 21.96 | 21.86 | 22.92 | 24.77 | 24.40 | 26.78 | 25.95 | 25.22 | 25.01 |
| 15 | 19.71 | 20.73 | 20.44 | 20.97 | 22.46 | 22.23 | 22.06 | 22.76 | 24.99 | 24.73 | 27.33 | 27.04 | 26.58 | 26.84 |
| 16 | 18.65 | 19.66 | 19.43 | 20.00 | 21.40 | 21.34 | 21.80 | 22.42 | 24.81 | 23.90 | 25.61 | 25.01 | 24.32 | 24.58 |
| 17 | 17.71 | 18.67 | 18.40 | 18.92 | 20.13 | 20.14 | 20.03 | 21.08 | 23.39 | 22.98 | 24.88 | 24.92 | 23.88 | 24.12 |
| 18 | 20.30 | 21.34 | 21.09 | 21.71 | 23.25 | 23.18 | 23.01 | 24.06 | 26.50 | 25.34 | 28.01 | 28.00 | 26.85 | 27.09 |
| 19 | 19.80 | 20.89 | 20.69 | 21.25 | 23.04 | 22.88 | 22.71 | 23.59 | 25.62 | 24.53 | 26.78 | 26.50 | 25.41 | 25.59 |
| 20 | 18.30 | 19.28 | 19.00 | 19.46 | 21.02 | 20.96 | 20.68 | 21.50 | 23.37 | 22.99 | 24.63 | 24.57 | 24.09 | 24.07 |
| Average | 18.95 | 19.97 | 19.63 | 20.16 | 21.65 | 21.57 | 21.49 | 22.33 | 24.59 | 24.10 | 26.18 | 25.69 | 24.99 | 25.25 |
| SD | 1.14 | 1.21 | 1.18 | 1.22 | 1.31 | 1.27 | 1.26 | 1.31 | 1.53 | 1.51 | 1.79 | 1.71 | 1.65 | 1.70 |

# Table S2-6. Body weight data of YHDM-treated mice during days 13-26****(g)****

| No. | d13 | d14 | d15 | d16 | d17 | d18 | d19 | d20 | d21 | d22 | d23 | d24 | d25 | d26 |
| --- | --- | --- | --- | --- | --- | --- | --- | --- | --- | --- | --- | --- | --- | --- |
| 1 | 20.98 | 21.33 | 21.54 | 21.98 | 24.15 | 23.72 | 24.00 | 24.38 | 29.48 | 29.45 | 31.48 | 30.47 | 29.45 | 31.87 |
| 2 | 22.03 | 22.44 | 22.69 | 23.04 | 25.40 | 24.97 | 25.39 | 25.67 | 30.51 | 30.32 | 32.44 | 31.43 | 30.37 | 32.73 |
| 3 | 19.33 | 19.76 | 19.94 | 20.39 | 22.55 | 22.34 | 22.76 | 22.92 | 28.15 | 28.30 | 30.41 | 29.30 | 28.51 | 30.74 |
| 4 | 20.30 | 20.74 | 20.96 | 21.42 | 23.67 | 23.65 | 23.70 | 24.21 | 29.67 | 29.88 | 32.36 | 31.13 | 30.14 | 32.80 |
| 5 | 20.92 | 21.27 | 21.48 | 21.87 | 23.97 | 23.31 | 23.59 | 23.77 | 29.65 | 29.57 | 31.28 | 30.31 | 29.08 | 31.55 |
| 6 | 22.14 | 22.65 | 22.92 | 23.42 | 25.89 | 25.06 | 25.26 | 25.82 | 31.01 | 31.02 | 32.87 | 31.80 | 30.74 | 32.64 |
| 7 | 20.85 | 21.25 | 21.40 | 21.77 | 23.85 | 23.30 | 23.63 | 24.33 | 29.58 | 29.31 | 31.10 | 30.08 | 29.01 | 31.39 |
| 8 | 19.94 | 20.37 | 20.45 | 20.84 | 22.66 | 22.39 | 22.59 | 22.80 | 27.48 | 27.39 | 29.16 | 28.33 | 27.16 | 29.37 |
| 9 | 19.39 | 19.80 | 19.98 | 20.49 | 22.75 | 22.51 | 21.76 | 21.89 | 26.99 | 26.96 | 28.67 | 27.76 | 26.60 | 29.01 |
| 10 | 20.15 | 20.53 | 20.73 | 21.08 | 23.40 | 22.88 | 22.83 | 23.09 | 28.75 | 28.65 | 30.43 | 29.29 | 28.50 | 30.59 |
| 11 | 19.12 | 19.41 | 19.66 | 19.78 | 21.83 | 21.80 | 22.13 | 22.43 | 26.48 | 26.27 | 27.59 | 26.68 | 25.97 | 27.82 |
| 12 | 21.06 | 21.61 | 21.89 | 22.33 | 24.82 | 24.18 | 24.38 | 24.58 | 30.55 | 30.51 | 31.87 | 31.10 | 30.30 | 33.12 |
| 13 | 22.58 | 23.03 | 23.36 | 23.91 | 26.52 | 25.64 | 26.63 | 26.94 | 33.21 | 33.16 | 36.13 | 34.56 | 33.50 | 36.31 |
| 14 | 21.77 | 22.13 | 22.29 | 22.79 | 24.89 | 23.99 | 24.24 | 24.85 | 30.78 | 30.15 | 31.90 | 31.55 | 30.67 | 33.34 |
| 15 | 20.46 | 20.91 | 21.03 | 21.51 | 23.69 | 23.04 | 23.31 | 23.49 | 28.99 | 28.76 | 30.32 | 29.24 | 28.36 | 30.42 |
| 16 | 19.33 | 19.72 | 19.82 | 20.31 | 22.55 | 22.36 | 22.81 | 23.37 | 28.55 | 28.54 | 31.04 | 29.98 | 28.90 | 31.55 |
| 17 | 20.54 | 20.86 | 21.01 | 21.51 | 23.22 | 23.29 | 23.57 | 23.86 | 29.05 | 28.99 | 31.05 | 31.61 | 30.34 | 32.57 |
| 18 | 21.02 | 21.44 | 21.58 | 22.15 | 24.09 | 23.48 | 24.16 | 24.65 | 29.54 | 29.69 | 31.54 | 30.56 | 29.95 | 32.00 |
| 19 | 22.00 | 22.50 | 22.72 | 23.18 | 25.96 | 25.71 | 26.13 | 26.19 | 32.32 | 32.32 | 35.15 | 33.01 | 31.49 | 33.84 |
| 20 | 20.43 | 20.70 | 20.87 | 21.34 | 23.31 | 23.07 | 23.57 | 23.98 | 28.74 | 28.89 | 31.29 | 30.03 | 28.76 | 30.73 |
| Average | 20.72 | 21.12 | 21.32 | 21.76 | 23.96 | 23.53 | 23.82 | 24.16 | 29.47 | 29.41 | 31.40 | 30.41 | 29.39 | 31.72 |
| SD | 1.02 | 1.05 | 1.08 | 1.12 | 1.28 | 1.11 | 1.27 | 1.29 | 1.65 | 1.64 | 1.94 | 1.78 | 1.72 | 1.88 |

# Table S2-7. Body weight data of YHDH-treated mice during days 13-26****(g)****

| No. | d13 | d14 | d15 | d16 | d17 | d18 | d19 | d20 | d21 | d22 | d23 | d24 | d25 | d26 |
| --- | --- | --- | --- | --- | --- | --- | --- | --- | --- | --- | --- | --- | --- | --- |
| 1 | 20.15 | 20.57 | 20.69 | 20.86 | 20.83 | 20.59 | 20.51 | 20.96 | 21.81 | 21.97 | 22.79 | 22.90 | 22.90 | 23.83 |
| 2 | 20.65 | 20.84 | 20.97 | 21.17 | 21.15 | 20.92 | 20.65 | 20.93 | 21.82 | 21.99 | 22.32 | 22.39 | 22.37 | 23.36 |
| 3 | 23.19 | 23.76 | 23.98 | 24.19 | 24.14 | 23.92 | 23.55 | 23.99 | 24.97 | 25.25 | 26.29 | 26.48 | 26.53 | 27.61 |
| 4 | 22.92 | 23.55 | 23.73 | 23.89 | 23.85 | 23.59 | 23.55 | 24.07 | 25.62 | 25.43 | 26.87 | 26.93 | 26.97 | 27.88 |
| 5 | 21.05 | 21.46 | 21.59 | 21.76 | 21.68 | 21.67 | 21.57 | 22.08 | 23.18 | 23.37 | 24.47 | 24.54 | 24.55 | 25.26 |
| 6 | 21.19 | 21.57 | 21.39 | 21.57 | 21.58 | 21.39 | 21.06 | 21.44 | 21.99 | 22.36 | 23.18 | 23.49 | 23.55 | 24.80 |
| 7 | 21.75 | 22.14 | 22.28 | 22.43 | 22.42 | 22.14 | 21.86 | 22.45 | 23.19 | 23.36 | 24.31 | 24.39 | 24.41 | 25.60 |
| 8 | 22.92 | 23.59 | 23.39 | 23.69 | 23.64 | 23.21 | 23.15 | 23.64 | 24.98 | 25.16 | 26.27 | 26.28 | 26.29 | 27.26 |
| 9 | 23.68 | 23.89 | 23.92 | 24.34 | 24.34 | 24.11 | 24.01 | 24.57 | 25.63 | 25.83 | 26.79 | 26.92 | 26.95 | 28.17 |
| 10 | 23.50 | 23.98 | 24.05 | 24.31 | 24.31 | 23.99 | 23.99 | 24.66 | 25.81 | 25.99 | 27.05 | 27.03 | 27.02 | 28.13 |
| 11 | 19.92 | 20.34 | 20.65 | 20.73 | 20.73 | 20.67 | 20.60 | 21.02 | 21.74 | 21.94 | 22.71 | 22.88 | 22.90 | 23.73 |
| 12 | 20.14 | 20.73 | 20.85 | 20.99 | 20.87 | 20.68 | 20.67 | 21.22 | 21.89 | 22.11 | 22.76 | 22.85 | 22.87 | 23.79 |
| 13 | 21.30 | 21.74 | 21.86 | 22.12 | 22.04 | 21.74 | 21.91 | 22.41 | 23.46 | 23.57 | 24.70 | 24.68 | 24.73 | 25.72 |
| 14 | 20.63 | 21.24 | 21.32 | 21.44 | 21.28 | 20.84 | 20.76 | 21.28 | 22.31 | 22.47 | 23.76 | 23.81 | 23.98 | 24.78 |
| 15 | 21.01 | 21.24 | 21.58 | 21.80 | 21.76 | 21.51 | 21.34 | 21.82 | 22.41 | 22.64 | 22.81 | 23.19 | 23.31 | 24.60 |
| 16 | 23.14 | 23.59 | 23.83 | 23.82 | 23.78 | 23.34 | 23.25 | 23.76 | 24.97 | 25.19 | 26.28 | 26.32 | 26.34 | 27.22 |
| 17 | 22.75 | 23.14 | 23.15 | 23.35 | 23.36 | 23.58 | 23.72 | 24.20 | 25.17 | 25.17 | 26.51 | 26.57 | 26.33 | 27.39 |
| 18 | 22.16 | 22.57 | 23.11 | 23.30 | 23.48 | 23.35 | 23.20 | 23.90 | 24.57 | 24.75 | 25.74 | 26.08 | 26.12 | 27.38 |
| 19 | 23.14 | 23.90 | 23.99 | 24.17 | 23.93 | 23.82 | 23.66 | 24.21 | 24.76 | 25.27 | 27.12 | 27.21 | 27.45 | 28.30 |
| 20 | 24.71 | 25.17 | 25.36 | 25.57 | 25.52 | 24.69 | 25.03 | 25.12 | 25.95 | 25.88 | 26.00 | 26.17 | 25.96 | 27.08 |
| Average | 21.99 | 22.45 | 22.59 | 22.78 | 22.74 | 22.49 | 22.40 | 22.89 | 23.81 | 23.99 | 24.94 | 25.06 | 25.08 | 26.09 |
| SD | 1.39 | 1.43 | 1.42 | 1.44 | 1.45 | 1.39 | 1.44 | 1.45 | 1.57 | 1.53 | 1.73 | 1.70 | 1.69 | 1.72 |

# Table S2-8. Body weight data of model mice during days 13-26****(g)****

| No. | d13 | d14 | d15 | d16 | d17 | d18 | d19 | d20 | d21 | d22 | d23 | d24 | d25 | d26 |
| --- | --- | --- | --- | --- | --- | --- | --- | --- | --- | --- | --- | --- | --- | --- |
| 1 | 18.10 | 18.88 | 17.67 | 18.74 | 18.83 | 17.59 | 16.19 | 18.97 | 19.13 | 17.95 | 20.12 | 20.03 | 18.39 | 17.34 |
| 2 | 18.94 | 19.75 | 18.57 | 19.68 | 19.70 | 18.57 | 17.75 | 19.99 | 20.15 | 18.96 | 21.46 | 21.28 | 19.60 | 18.73 |
| 3 | 19.35 | 20.19 | 18.92 | 20.06 | 20.13 | 18.84 | 17.75 | 20.29 | 20.48 | 19.23 | 21.61 | 21.44 | 19.71 | 18.66 |
| 4 | 19.11 | 19.94 | 18.68 | 19.81 | 19.82 | 18.55 | 17.24 | 19.85 | 20.05 | 18.82 | 21.01 | 21.07 | 19.39 | 18.32 |
| 5 | 18.18 | 18.98 | 17.87 | 18.92 | 18.95 | 17.75 | 16.72 | 19.07 | 19.24 | 18.02 | 21.07 | 20.81 | 19.18 | 18.32 |
| 6 | 20.07 | 20.94 | 19.73 | 20.89 | 20.91 | 19.86 | 18.84 | 21.24 | 21.36 | 20.18 | 22.98 | 22.66 | 20.89 | 19.84 |
| 7 | 20.23 | 21.23 | 19.83 | 21.23 | 21.30 | 19.92 | 18.59 | 21.48 | 21.66 | 20.34 | 22.91 | 22.87 | 21.05 | 19.93 |
| 8 | 19.70 | 20.56 | 19.35 | 20.50 | 20.56 | 19.24 | 18.34 | 20.91 | 21.03 | 19.77 | 22.38 | 22.25 | 20.47 | 19.49 |
| 9 | 18.30 | 19.08 | 17.58 | 18.74 | 18.78 | 17.52 | 16.23 | 18.86 | 19.05 | 17.77 | 20.01 | 19.93 | 18.32 | 17.33 |
| 10 | 18.97 | 19.82 | 19.10 | 20.00 | 20.02 | 18.68 | 17.71 | 20.10 | 20.25 | 19.03 | 21.48 | 21.35 | 19.67 | 18.80 |
| 11 | 20.09 | 20.93 | 19.88 | 20.98 | 21.00 | 19.75 | 18.84 | 21.21 | 21.31 | 20.05 | 22.44 | 22.26 | 20.50 | 19.52 |
| 12 | 20.76 | 21.66 | 20.47 | 21.68 | 21.73 | 20.34 | 19.19 | 21.87 | 22.05 | 20.74 | 23.19 | 23.06 | 21.22 | 20.30 |
| 13 | 18.49 | 19.29 | 18.30 | 19.29 | 19.30 | 18.28 | 17.54 | 19.52 | 19.63 | 18.47 | 20.93 | 20.76 | 19.14 | 18.30 |
| 14 | 19.53 | 20.16 | 19.00 | 19.94 | 19.97 | 18.49 | 17.59 | 20.12 | 20.27 | 19.05 | 21.50 | 21.38 | 19.67 | 18.81 |
| 15 | 18.24 | 19.08 | 17.72 | 18.94 | 19.06 | 17.70 | 16.26 | 19.12 | 19.45 | 18.27 | 20.34 | 20.23 | 18.63 | 17.77 |
| 16 | 18.68 | 19.53 | 18.18 | 19.44 | 19.49 | 18.24 | 17.16 | 19.66 | 19.90 | 18.69 | 21.05 | 20.92 | 19.25 | 18.39 |
| 17 | 18.22 | 19.08 | 17.77 | 18.92 | 19.14 | 17.87 | 16.96 | 19.27 | 19.44 | 18.28 | 20.47 | 20.35 | 18.79 | 17.83 |
| 18 | 18.73 | 19.53 | 18.36 | 19.43 | 19.48 | 18.20 | 17.12 | 19.63 | 19.82 | 18.59 | 20.60 | 20.64 | 18.93 | 17.85 |
| 19 | 19.81 | 20.66 | 19.51 | 20.67 | 20.69 | 19.40 | 18.29 | 20.88 | 21.03 | 19.73 | 21.95 | 21.82 | 20.08 | 18.98 |
| 20 | 21.04 | 21.91 | 20.71 | 21.83 | 21.83 | 20.56 | 19.70 | 22.14 | 22.20 | 20.86 | 23.43 | 23.22 | 21.34 | 20.33 |
| Average | 19.23 | 20.06 | 18.86 | 19.99 | 20.03 | 18.77 | 17.70 | 20.21 | 20.37 | 19.14 | 21.55 | 21.42 | 19.71 | 18.74 |
| SD | 0.90 | 0.93 | 0.95 | 0.97 | 0.96 | 0.94 | 1.01 | 1.00 | 0.98 | 0.94 | 1.05 | 1.02 | 0.94 | 0.92 |
